# Cranberry improves metabolic syndrome-related organ dysfunction in rats by modulating AMPK/SREBP1, ROCK1 and TGF-β1

**DOI:** 10.1038/s41598-025-16925-2

**Published:** 2025-09-15

**Authors:** Sahar M. Elashmony, Yosra Alhindi, Dina H. Merzeban, Rehab A. Mohammed, Asmaa Mohamed Elsayed, Marwa A. Sofi, Rania H. Mahmoud, Hanan A. Shamardl, Dina Elsayed Shaker

**Affiliations:** 1 Medical Pharmacology Department, Faculty of Medicine, Cairo University, Cairo, Egypt; 2 Pharmacology and Toxicology Department, Faculty of Medicine, Umm Al-Qura University, Makkah, Saudi Arabia; 3 Medical Physiology Department, Faculty of Medicine, Fayoum University, Fayoum, Egypt; 4 Histology and Cell Biology Department, Faculty of Medicine, Fayoum University, Fayoum, Egypt; 5 Medical Biochemistry and Molecular Biology Department, Faculty of Medicine, Fayoum University, Fayoum, Egypt; 6 Medical Pharmacology Department, Faculty of Medicine, Fayoum University, Fayoum, 19052 Egypt

**Keywords:** Cranberry extract, Metabolic syndrome, AMPK/*Srebf1*, *Rock1*, TGF-β1, Biochemistry, Drug discovery, Molecular biology, Physiology

## Abstract

Metabolic syndrome (MetS) is a widespread, complex health issue that poses a substantial global health burden with increased healthcare costs and reduced quality of life, necessitating effective prevention and management strategies. This study aimed to investigate the potential therapeutic effects of cranberry extract (*Vaccinium macrocarpon*) and metformin on metabolic syndrome in a rat model. Forty rats were divided into the following groups: normal control, MetS (high fat and fructose for 4 weeks followed by streptozotocin 35 mg/kg, i.p.), MetS + cranberry (50 mg/kg), MetS + cranberry (100 mg/kg), and MetS + metformin (200 mg/kg) groups. Treatments were given orally for four weeks with the continuation of a high-fat and high-fructose diet. The evaluations included key metabolic parameters, liver and kidney pathology, and relevant molecular pathways. The present results revealed that MetS induction significantly increased body weight, BMI, fasting glucose, and OGTT results; impaired lipid profile, creatinine and blood pressure; and upregulated hepatic gene expression of Rho-associated protein kinase 1 (*Rock1* ) and sterol regulatory element-binding transcription factor 1 (*Srebf1*), which encodes the protein SREBP-1c. In addition to hepatic and renal structural abnormalities, increased collagen and increased iNOS/TGF-β1 immunoreactivity were observed. Cranberry ameliorated metabolic parameters in a dose-dependent manner, upregulated adenosine monophosphate-activated protein kinase (AMPK), downregulated *Rock1* and Srebf1 expression, improved the histopathology of the liver and kidney and decreased the immunoexpression of iNOS and TGF-β1. The results for cranberry were generally comparable to those for metformin. In conclusion, cranberry extract is potentially a safe therapeutic strategy for MetS, offering broad-spectrum action, organ protection, and molecular pathway modulation. These findings strongly support cranberry as a promising natural approach for managing MetS.

## Introduction

Metabolic syndrome (MetS), a global health crisis characterized by central obesity, hyperglycemia, dyslipidemia, and hypertension, is a major driver of chronic disease burden worldwide. Its alarming prevalence, fueled by modern sedentary lifestyles and obesogenic diets, significantly elevates the risk of developing type 2 diabetes, cardiovascular disease, nonalcoholic fatty liver disease (NAFLD), and chronic kidney disease, impacting both lifespan and healthspan^1^.

While metformin is a highly effective and widely prescribed first-line therapy for managing metabolic syndrome and type 2 diabetes, its clinical utility can be limited by well-documented adverse effects. Most notably, a significant portion of patients experience gastrointestinal intolerance, such as diarrhea and nausea, which often leads to poor adherence or discontinuation of the drug^2^. Furthermore, metformin is contraindicated in patients with severe renal or hepatic disease due to the risk of lactic acidosis^3^therefore effective and safe therapeutic alternatives is needed.

In this context, natural products, particularly those rich in bioactive polyphenols, have emerged as promising candidates for MetS management. Cranberry (*Vaccinium macrocarpon*), a widely consumed berry, is a potent source of diverse polyphenolic compounds, including anthocyanins, proanthocyanidins, and flavonoids^4^which are known for their antioxidant and anti-inflammatory properties^55^. Preclinical studies have consistently demonstrated the beneficial effects of cranberry and cranberry extracts on key metabolic parameters. For example, studies have shown that cranberries can improve glucose tolerance and insulin sensitivity in animal models of diabetes^7^modulate dyslipidemia^8^and mitigate oxidative stress (OS) and systemic inflammation^5^.

Hepatic dysfunction is a central feature of MetS, with dysregulated lipid metabolism, inflammation, and OS leading to nonalcoholic fatty liver disease (NAFLD)^9^. Crucially, dysregulation of hepatic signaling pathways plays a critical role in this pathology. Elevated hepatic Rho-associated protein kinase 1 (ROCK1) expression and activity have been linked to increased inflammation and fibrosis in NAFLD models^10^. Conversely, adenosine monophosphate-activated protein kinase (AMPK) activation in the liver promotes glucose and lipid metabolism^11^reduces inflammation, and protects against hepatic steatosis^12^. Sterol regulatory element-binding protein-1c (SREBP-1c), a key regulator of lipogenesis, is often overexpressed in NAFLD, driving excessive hepatic lipid accumulation^13^.

Studies using animal models of NAFLD and diabetic nephropathy have demonstrated that polyphenol-rich extracts can improve hepatic and renal damage^14^. Furthermore, interventions that lead to reductions in excessive collagen deposition, and the downregulation of proinflammatory mediators, such as iNOS (which is often upregulated under pathological conditions leading to increased oxidative stress and inflammation)^15^and profibrotic markers such as transforming growth factor-β1 (TGF-β1)^16^ have a protective effect against organ fibrosis and inflammation.

However, while existing research provides some rationale, a comprehensive in vivo evaluation of cranberry’s effects on hepatic and renal tissues, particularly in direct comparison to a standard treatment such as metformin, is still needed to test its therapeutic potential for MetS-related organ protection.

Therefore, our study aimed to investigate the therapeutic efficacy of cranberry extract (*Vaccinium macrocarpon*) in a rat model of metabolic syndrome to provide valuable insights into the translational potential of cranberry as a natural, multitarget therapeutic agent for metabolic syndrome and its associated organ complications.

## Materials and methods

### Experimental animals

Forty male Wistar rats aged one month and weighing 130–150 g were used in this study. The animals were maintained under conventional laboratory conditions with a temperature of 20 ± 5 °C and a regular 12-h light/dark cycle.

### Ethics statement

The experimental protocol was approved by the ethics committee of research, Faculty of Medicine, Fayoum University (R655), and all procedures adhered to the ARRIVE guidelines.

### Drugs

Cranberry extract (Vaccinium Macrocarpon, 1000 mg/ml) was obtained from Commerce Drive (Hauppauge, NY, 11688, USA). While a detailed phytochemical analysis of the specific batch used was not performed within this study, Vaccinium macrocarpon is well-documented in the literature as a rich source of diverse bioactive polyphenolic compounds. These key constituents, primarily include A-type proanthocyanidins (PACs), anthocyanins (e.g., cyanidin, peonidin glycosides), flavonols (e.g., quercetin, myricetin), phenolic acids, and triterpenoids such as ursolic acid^17 4 5 6^. For administration, the cranberry extract was diluted in distilled water and given orally by gavage.

Metformin tablets were crushed, 2 drops of tween were added to make a paste to ensure better dissolution in distilled water and given orally.

### Experimental design

Beginning on day 0, animals were divided into 5 groups: the normal control group (*n* = 8) was fed a standard chow diet composed of (20% protein, 60% carbohydrates, 6% fat and 10% fibers) and the metabolic syndrome groups (*n* = 32). The MetS group was fed a high fat/high fructose (HF/HF) diet composed of (25% margarine fat in the diet added to standard diet and 25% fructose in the drinking water)^18 19^ for eight weeks. On day 28, single dose of intraperitoneal injection of streptozotocin (STZ) (35 mg/kg body weight dissolved in 0.01 M citrate buffer (pH = 4.5))^20^. Both HF/HF diet and the low doses of STZ are essential elements to induce T2DM with insulin resistance as a part of metabolic syndrome^18 21^.

The development of metabolic syndrome was confirmed by a combination of factors: a body mass index (BMI) > 0.55 g/cm² (a marker for obesity in rats)^22^increased systolic and diastolic blood pressure, and fasting blood glucose levels > 200 mg/dl. After induction, the treatment groups received their respective interventions daily for four weeks with the continuation of the HF/HF diet.

### Experimental groups

The rats were divided into five groups (eight rats each): (Group 1) *the normal control* group; (Group 2): *the MetS group*: given HF/HF diet for 8 weeks as prescribed above (Group 3): *the MetS + cranberry 50* mg/kg/day orally by gavage for 4 weeks group; (Group 4): *the MetS + cranberry 100* mg/kg/day orally by gavage for last 4 weeks group; [The doses of Cranberry were selected based on a pilot study as well as a previous study^23^in addition the cranberry extract doses (50 and 100 mg/kg/day) are equivalent to human doses (HEDs) of approximately 570–1130 mg/day for a 70 kg adult, using body surface area normalization (HED (mg/kg) = Animal dose (mg/kg) × [Animal Km / Human Km])^24^. This range aligns with some commercial cranberry supplements, suggesting clinical feasibility and (Group 5): *the MetS + metformin group*, which received metformin at 200 mg/kg/day^25^ orally by gavage for the last 4 weeks. This dosage was selected as it is commonly used in rodent models of metabolic syndrome and corresponds to the upper therapeutic range in humans (approximately 2270 mg/day for a 70 kg adult) when converted using body surface area normalization. This ensures a robust positive control for comparing therapeutic efficacy.

#### Anthropometric measurements

Anthropometric measurements, including body weight and body length, BMI was calculated as weight (g)/length (cm2) (g/cm2). These measurements were done on day 28 for confirmation of obesity for further induction of MetS by streptozotocin, and on the day of dissection for the final results.

#### Blood pressure measurement

Blood pressure was measured weekly via a computerized noninvasive blood pressure system (ML 125 NIBP, AD Instruments, Australia), which employs the volume pressure method to record systolic (SBP) and diastolic (DBP) blood pressure values. Mean arterial blood pressure (MAP) was calculated as MAP = diastolic + 1/3pulse pressure.

#### Oral glucose tolerance test (OGTT)

An oral glucose load of 2 gm/kg body weight was given following overnight fasting for 12 h. Blood samples were collected from the tail vein at 0 (just before the glucose load), 30, 60, 90, and 120 min after glucose intake for measurement of blood glucose levels^26^.

#### Euthanasia and tissue sampling

Following the completion of the experimental period, the rats were humanely euthanized to ensure minimal suffering and distress. Euthanasia was performed by first inducing deep anesthesia in the rats with phenobarbitone (dose 30 mg/kg, IP)^27^followed 10 min later by cervical dislocation to ensure humane and rapid death. All animal procedures, including euthanasia, were performed in strict accordance with the guidelines approved by the ethics committee of research, Faculty of Medicine, Fayoum University (R655), and all procedures adhered to the ARRIVE guidelines.

Intracardiac blood samples were collected, the samples were centrifuged, and the serum was separated for biochemical measurements. Specimens from the liver and kidney were promptly excised. A portion of each tissue sample was preserved in 10% neutral formalin for histopathological and immunohistochemical examination. The remaining tissue samples were homogenized in 0.1 M phosphate buffer (pH 7.4) to obtain 20% w/v tissue homogenates, which were subsequently centrifuged, and the supernatants were collected and preserved at -70 °C for further biochemical measurements.

#### Biochemical marker assessment

Total cholesterol (TC), triglyceride (TG), HDL-cholesterol (HDL-C) (Vitro Scient., Egypt), ALT (Spinreact, Egypt) and creatinine (Diamond, Egypt) were quantified in the serum samples via the colorimetric method. Serum VLDL-C and LDL-C levels were calculated via the Friedewald formula: VLDL-C = TG/5 and LDL-C = TC - HDL-C - TG/5^28^.

#### Quantitative real-time-PCR (qRT‒PCR) analysis

Total RNA was isolated from liver tissue homogenates via a Qiagen tissue extraction kit (Qiagen) according to the manufacturer’s instructions. The concentrations of the RNA samples were measured via spectrophotometry (dual wavelength Beckman, Spectrophotometer, USA). One microgram of RNA was used for cDNA production for reverse transcription‒PCR via the Excel RTTM One-step RT‒qPCR Kit as described in the manufacturer’s protocol (Smobio Technology, Inc., Taiwan).

qRT‒PCR was carried out via Macrogen gene expression assays (Macrogen, Inc., Korea) for rho-associated coiled coil containing protein kinase-1 (ROCK-1), sterol regulatory element binding protein-1c (Srebf1), AMP-activated protein kinase (AMPK) and B-actin. The sequences of the primers used were as follows: for *Rock1*, forward primer 5ʹ-ATG CCA TGT TAA GTG CCA CA-3ʹ and reverse primer 5ʹ-AAC CAG AAG GTG GGT TCTT-3ʹ. For Srebf1: forward primer 5ʹ - GTGGTCTTCCAGAGGCTGAG − 3ʹ, reverse primer: 5ʹ - GGG TGA GAG CCT TGA GAC AG -3ʹ; for AMPK: forward primer 5ʹ- AGC TCG CAG TGG CTT ATC AT -3ʹ, reverse primer: 5ʹ - GGG GCT GTC TGC TAT GAG AG -3ʹ; and for B-actin: forward primer 5ʹ - GAT ATC GCT GCG CTC GTC − 3ʹ, reverse primer: 5ʹ - TGG GGT ACT TCA GGG TCA GG-3ʹ. Finally, real-time RT‒PCR was performed in a 20-µl reaction volume consisting of 10 µl of 2X SYBR Green PCR mix, 0.5 µl of each primer and 2 µl of cDNA. Thermal cycling conditions were used, including a preamplification step at 95 °C for 1 min, followed by amplification for 40 cycles at 95 °C for 15 s and 60 °C for 1 min. Changes in the expression of the studied genes were demonstrated via the threshold cycle (CT) method. All values were measured as the ratio of each gene to B-actin, which was used as a housekeeping gene, via the ΔΔCt method^29^. The relative value for the control group was detected as one.

### Tissue processing and histological assessment

Liver and kidney tissues preserved in a 10% formalin solution underwent standard histological processing before being embedded in paraffin. Subsequently, 5-micrometer-thick sections were prepared via a microtome. These sections were then stained with hematoxylin and eosin (H&E) according to established protocols for general histological examination and assessment of tissue morphology. Periodic acid–Schiff (PAS) histochemistry technique (to demonstrate glycogen accumulation) and Masson’s trichrome staining (for collagen fibers detection) were carried out as well^30^.

### Immunohistochemical procedures

Immunohistochemical reaction using anti-Inducible nitric oxide synthase (iNOS) rabbit polyclonal antibodies (Abcam, USA; Catalog NO: ab3523 at 1/200 dilution) and anti-Transforming growth factor (TGF-β1) rabbit monoclonal antibodies (Abcam, USA; Catalog NO: ab215715 at 1/500 dilution). liver and kidney samples were overnight incubated at 4 ◦C, followed by a reaction with biotinylated secondary antibody. After streptavidin peroxidase conjugation, 3,3- diaminobenzidine (DAB) was used as a chromogen and tissues were counterstained with hematoxylin. Excluding of the primary antibodies was done to obtain negative controls. Positive control of iNOS was human heart, while positive control of TGF-β1 was rat spleen (as provided by the manufacturer). The reaction of both iNOS and TGF-β1 was cytoplasmic.

### Quantitative image analysis

To quantify the histological and immunohistochemical findings, we performed morphometric analysis via the ToupView image analysis system (China). To assess the area percentage of PAS reaction, collagen fibers deposition, iNOS and TGF-β immunoexpression levels in liver and kidney tissues. This was achieved by analyzing ten randomly chosen, non overlapping microscopic fields per slide at ×100 magnification. The assessments were performed by an investigator blinded to the experimental group assignments to ensure objectivity and mitigate potential observer bias in the interpretation of the results.

### Statistical evaluation

Statistical analyses were carried out via SPSS software, version 18.0. Data are presented as the mean and standard deviations (S.D.). Area under the curve (AUC) values was determined as mean and standard error using the trapezoidal rule for time-course data. To determine statistically significant differences between the experimental groups, one-way analysis of variance (ANOVA) was performed, and posthoc pairwise comparisons were conducted via Tukey’s test. A p value of less than 0.05 was predetermined as statistically significant.

## Results

### Effects on body weight, BMI, and absolute and relative organ weights

**Fig. 1:**
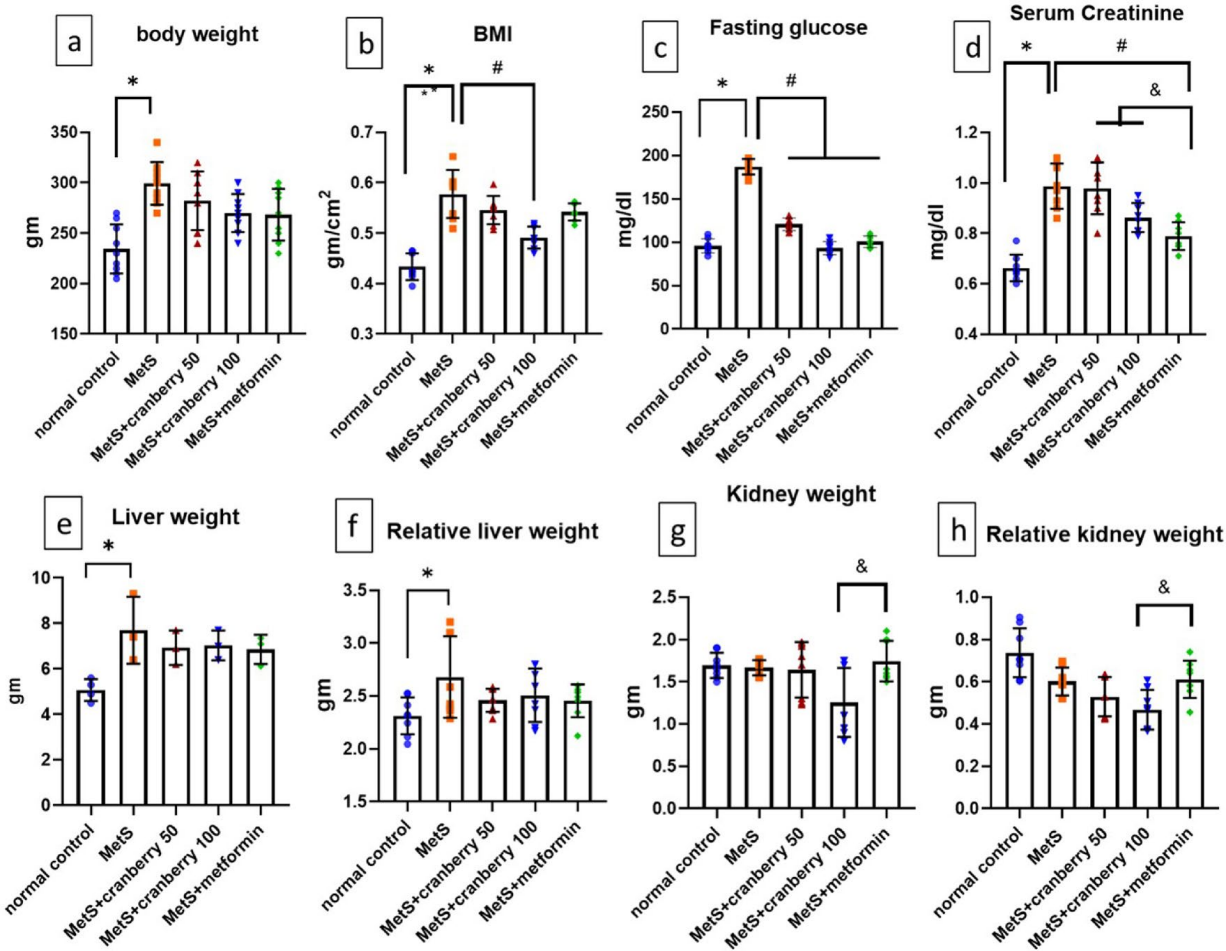
Means and standard deviations of (**a**) body weight and organ weight (gm), (**b**) BMI (gm/cm2), (**c**) fasting glucose level (mg/dl), (**d**) serum creatinine level (mg/dl) and (**e**–**h**) organ weight (gm) in all the studied groups. *Significant compared with the normal control group; ^#^significant compared with the MetS group; ^&^significant compared with the metformin group. Values of *p* < 0.05 were considered significant.

Body weight and BMI were significantly greater (*P* < 0.05) in the MetS group than in the normal control group. Compared with the MetS group, all the therapeutic interventions (cranberries 50 and 100 and metformin) significantly decreased (*P* < 0.05) body weight and BMI, with nonsignificant differences between the treatment groups, as shown in Fig. 1a, b. The absolute and relative weights of both the liver and the kidney were not significantly different across all groups except for the absolute and relative kidney weights, which were significantly lower (*P* < 0.05) in the cranberry 100 treatment group than in the metformin-treated group (Fig. 1e, f,g, h).

### Effects on fasting glucose levels and the glucose tolerance test

Compared with those in the normal control group, fasting glucose levels significantly increased (*P* < 0.05) in the MetS group. Compared with the MetS group, all the therapeutic interventions (cranberries 50 and 100 and metformin) significantly decreased (*P* < 0.05) fasting glucose levels, with insignificant differences between the treatment groups, as shown in Fig. 1c.

**Fig. 2:**
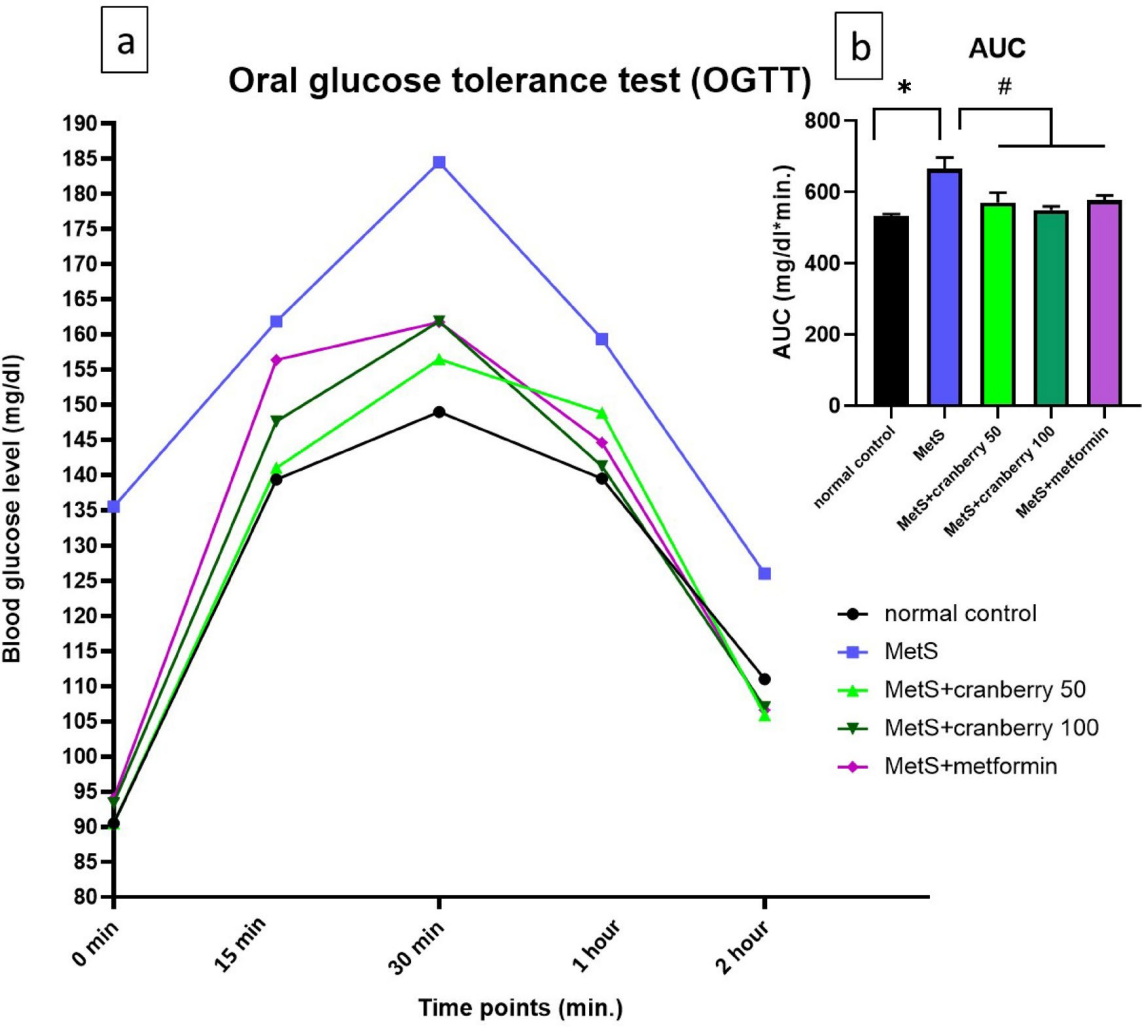
Effect of cranberry extract and metformin on oral glucose tolerance test (OGTT) and glucose area under the curve (AUC). (**a**) Blood glucose levels (mg/dl) at 0, 15, 30, 60, and 120 min after an oral glucose load. (**b**) Integrated area under the curve (AUC) for glucose (mg/dl*min.) derived from the OGTT data. Groups shown are: normal control (black circle), MetS (blue square), MetS + cranberry 50 mg/kg (green upward triangle), MetS + cranberry 100 mg/kg (dark green downward triangle), and MetS + metformin (purple diamond). Data are presented as mean values and standard errors. *Significant compared to normal control group; ^#^Significant compared to MetS group. Values of *p* < 0.05 were considered significant.

Furthermore, the results of oral glucose tolerance test (OGTT) showed that blood glucose levels in the MetS group rose sharply, peaking at 30 min and remained significantly elevated throughout the 2-hour test period compared to the normal control group. Treatment with cranberry extract at both 50 mg/kg and 100 mg/kg, as well as metformin, notably attenuated the rise in blood glucose levels compared to the untreated MetS group. Specifically, the peak glucose levels and the overall glucose excursion were visibly reduced in these treatment groups, with profiles appearing more similar to the normal control group, particularly at later time points as shown in Fig. 2a.

To quantify the overall glycemic response, the area under the curve (AUC) for glucose was calculated as shown in Fig. 2b. The MetS group exhibited a significantly higher AUC compared to the normal control group (*p* < 0.05). Importantly, administration of cranberry extract at 50 mg/kg, 100 mg/kg, and metformin resulted in a significant reduction in glucose AUC compared to the MetS group (*p* < 0.05). There were no statistically significant differences observed in AUC values among the intervention groups.

### Effect on systolic and diastolic blood pressure

**Fig. 3:**
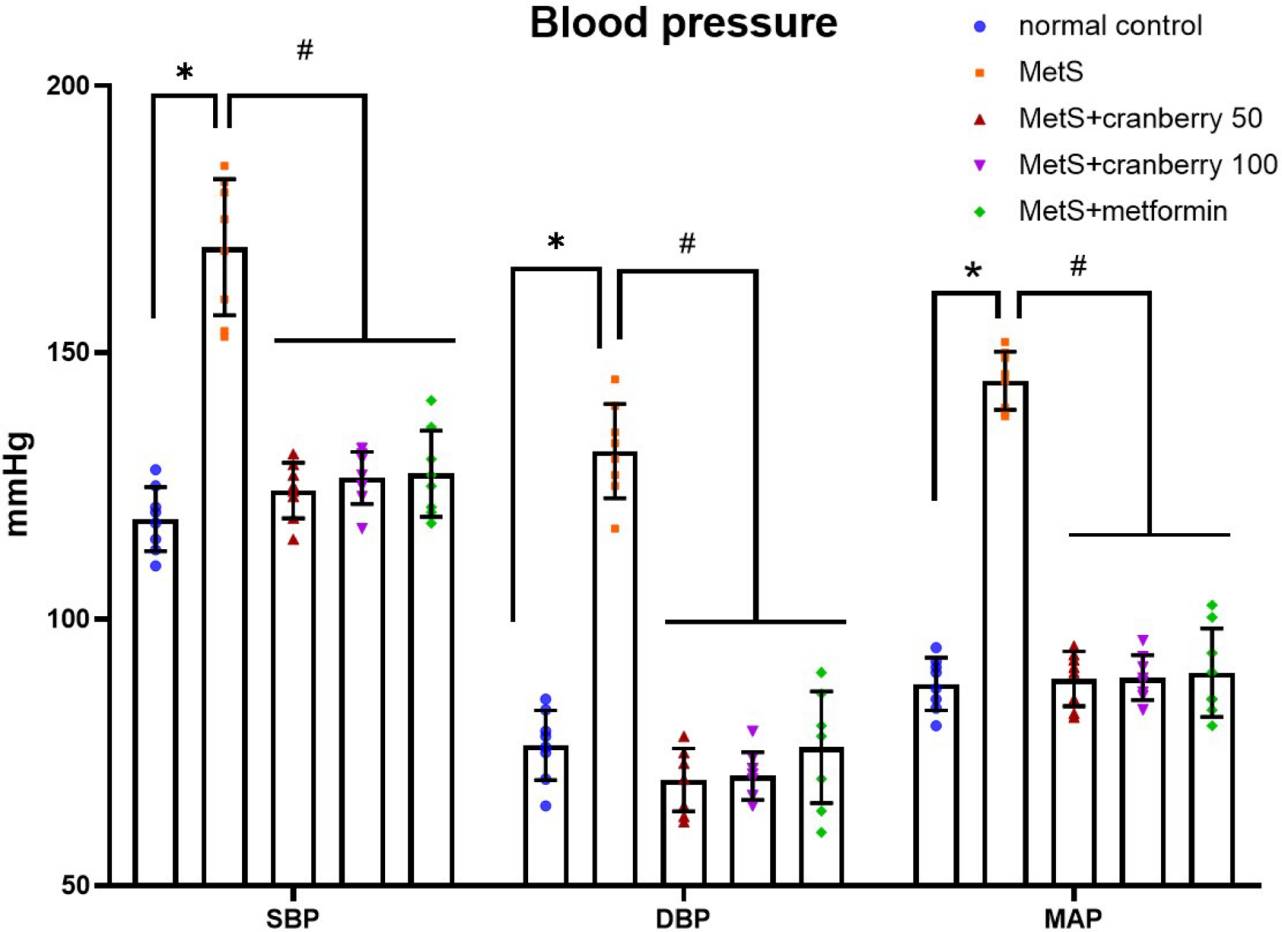
Means and standard deviations of systolic (SBP), diastolic (DBP) blood pressure and mean arterial blood pressure (MAP) (mmHg) in all the studied groups. *: significant compared with the normal control group; #: significant compared with the MetS group. Values of *p* < 0.05 were considered significant.

Analysis of the blood pressure results revealed that the MetS group presented significantly increased systolic (SBP), diastolic (DBP) and mean arteial blood pressure (MAP) compared with the normal control group (*P* < 0.05). Compared with the MetS group, treatment with both cranberry extracts (50 and 100) and metformin significantly decreased SBP, DBP and MAP (*P* < 0.05). There were no significant differences between the treatment groups (cranberry 50, cranberry 100, and metformin), as shown in Fig. 3.

### Effect of cranberries on the serum lipid profile

**Fig. 4:**
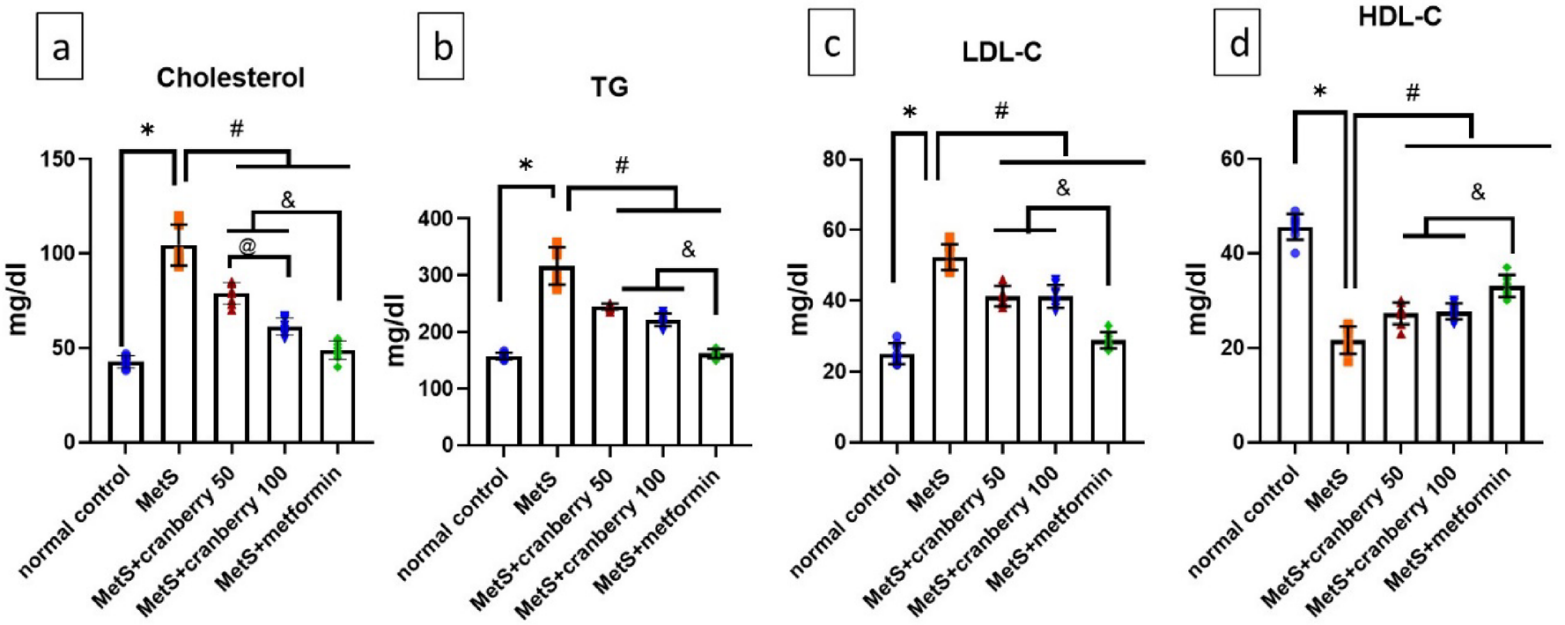
Means and standard deviations in mg/dl of (**a**) triglycerides (TG), (**b**) cholesterol, (**c**) low-density lipoprotein-cholesterol (LDL-C), and (**d**) high-density lipoprotein-cholesterol (HDL-C) in all the studied groups. *: significant compared with the normal control group; #: significant compared with the MetS group; @: significant compared with the cranberry 50 group; &: significant compared with the metformin group. Values of *p* < 0.05 were considered significant.

Analysis of the lipid profile revealed that the MetS group presented significantly increased levels of total cholesterol, triglycerides (TGs), and LDL-C, whereas HDL-C was significantly lower (*P* < 0.05) in the MetS group than in the normal control group. Compared with MetS, treatment with both cranberry extracts (50 and 100) and metformin significantly reversed these alterations (*P* < 0.05), with metformin showing significant improvement (*P* < 0.05) compared with cranberry treatments. In addition, cranberries had a dose-dependent effect on total cholesterol (*P* < 0.05), as shown in Fig. 4a–d.

### Effects on *Rock1*, AMPK and *Srebf1* gene expression

**Fig. 5:**
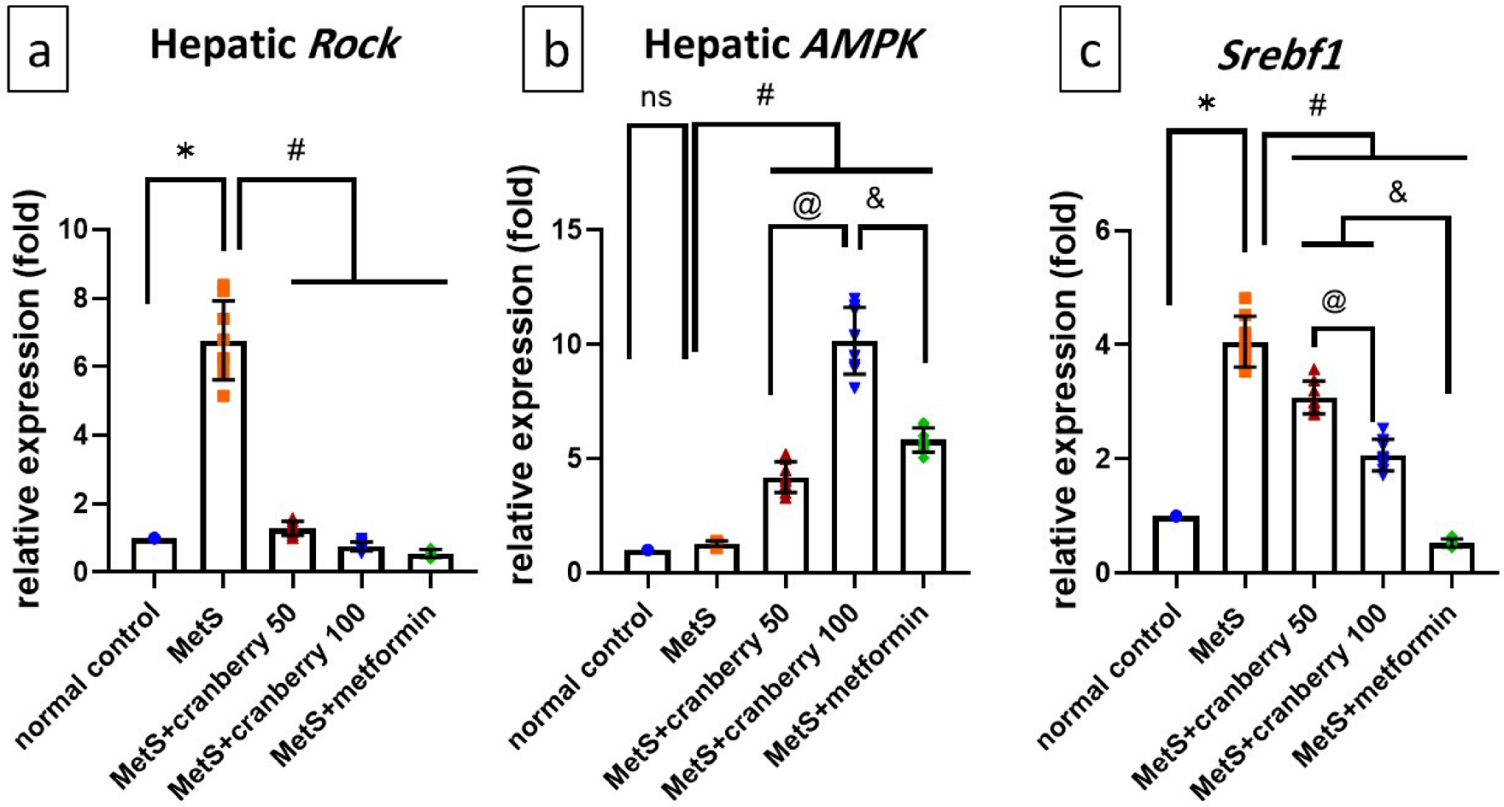
Means and standard deviations of the relative fold changes in the expression of (**a**) Rock1, (**b**) AMPK, and (**c**) Srebf1 in all the studied groups. ns: non-significant, *: significant difference compared with the normal control group; #: significant difference compared with the MetS group; @: significant difference compared with the cranberry 50 group; &: significant difference compared with the metformin group. Values of *p* < 0.05 were considered significant.

Analysis of the molecular markers revealed distinct expression patterns. Hepatic *Rock1* expression was significantly greater in the MetS group than in the normal control group (*P* < 0.05). Compared with MetS, all the treatments significantly decreased *Rock1* expression (*P* < 0.05), with nonsignificant differences between the treatment groups, as shown in Fig. 5a.

Although hepatic AMPK in the MetS group did not significantly differ from that in the normal control group, both the cranberry treatment group and the metformin group presented significantly increased (*P* < 0.05) AMPK expression compared with that in the MetS group. Notably, the level of AMPK was significantly greater (*P* < 0.05) in cranberry 100 than in cranberry 50 and metformin (Fig. 5b).

In terms of the expression of hepatic sterol regulatory element-binding transcription factor 1 (*Srebf1*), which encodes the protein sterol regulatory element-binding protein-1c (SREBP-1c), the MetS group presented significantly increased Srebf1 expression compared with the normal control group (*P* < 0.05). Cranberry extracts (50 and 100) and metformin significantly suppressed Srebf1 expression (*P* < 0.05) compared with MetS. Notably, metformin demonstrated superior effectiveness, as it significantly suppressed Srebf1 expression compared with that of the cranberry 50 and 100 treatments (*P* < 0.05). However, cranberries still show dose-dependent suppression of Srebf1, with cranberry 100 showing significantly greater suppression of Srebf1 than cranberry 50.

### Effects on the serum creatinine level

Compared with the normal control group, the MetS group presented significantly elevated serum creatinine levels (*P* < 0.05). Compared with MetS, treatment with 50 or 100 mg/kg cranberry or metformin significantly decreased the serum creatinine level (*P* < 0.05) (Fig. 1d).

To summarize the biochemical findings, the MetS group developed significant weight gain, hyperglycemia, hypertension, dyslipidemia, and elevated creatinine. These pathological changes were significantly attenuated by both cranberry and metformin. These improvements were accompanied by a significant modulation of key hepatic signaling pathways: the expression of pro-fibrotic ROCK1 and lipogenic SREBP-1c was suppressed, while the metabolic regulator AMPK was upregulated in the treated groups compared to the MetS group.

## Histological and immunohistochemical results

### Hepatic histopathology

**Fig. 6:**
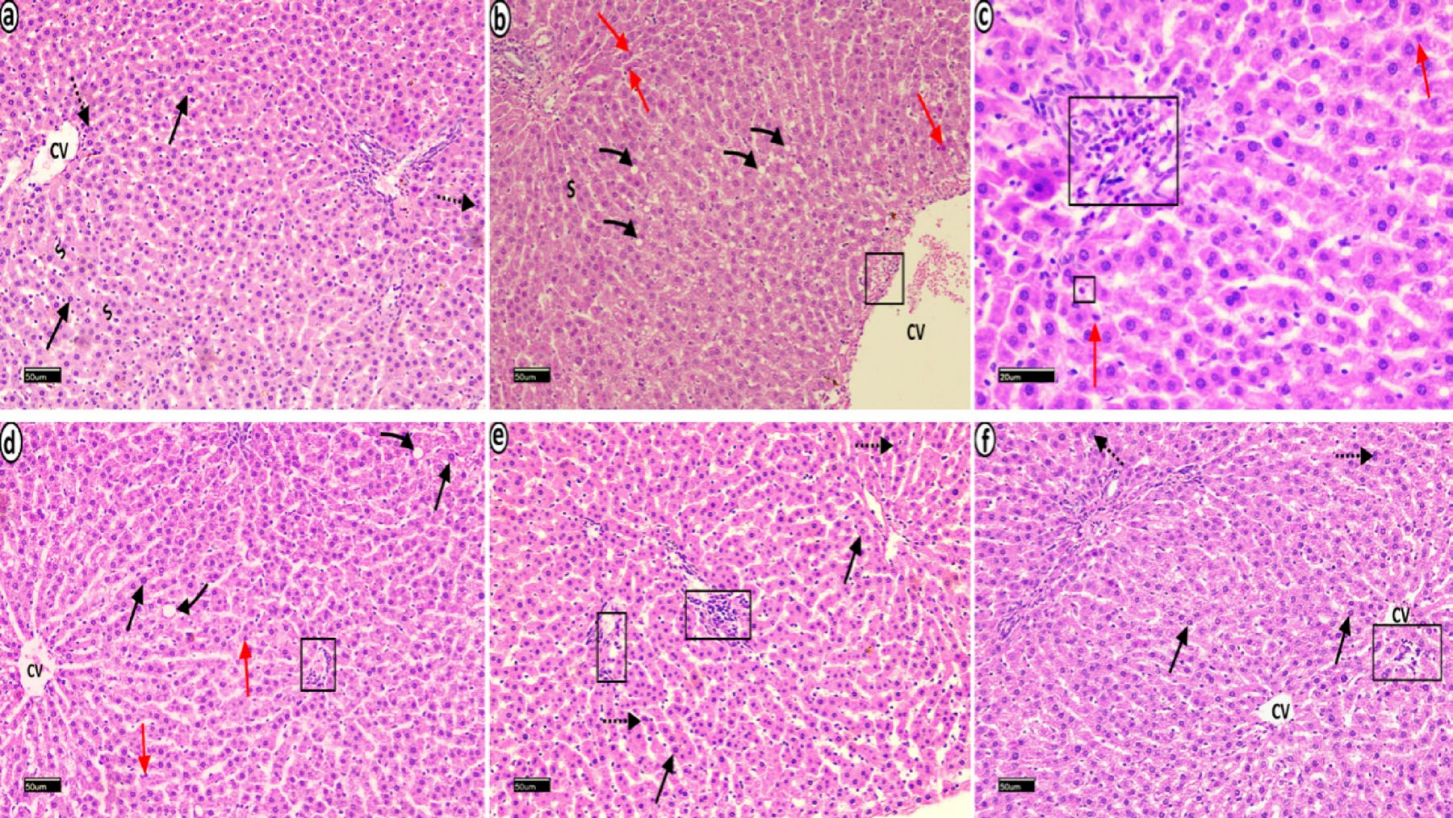
Photomicrographs of H&E-stained liver sections from all experimental groups: Control group (**a**) showing normal histological architecture. Hepatocytes are arranged in radiating cords extending from the central vein (CV) and separated with blood sinusoids (S). Hepatocytes display granular acidophilic cytoplasm and vesicular nuclei with prominent nucleoli (arrows). Some hepatocytes are binucleated (dotted arrows). MetS group (**b**,**c**) showing numerous hepatocytes with cytoplasmic vacuolations (curved arrows). The other hepatocytes presents deep acidophilic cytoplasm and small dark pyknotic nuclei (red arrows). A dilated central vein with thickened walls (CV) and prominent inflammatory cell infiltration are observed (black rectangles). The MetS + Cranberry 50 group (**d**) shows almost normal hepatocytes with vesicular nuclei (arrows). However, few hepatocytes display small, dark pyknotic nuclei (red arrows) or cytoplasmic vacuolations (curved arrows). Mild inflammatory infiltration (black rectangle) is evident. The MetS + Cranberry 100 (**e**) and metformin (**f**) groups present restored normal hepatic architecture (arrows) with some binucleated cells (dotted arrows). Minimal inflammatory cells are observed (black rectangles). (H&E **a**,**b**,**d**,**e**,**f** x200 c x400)

H&E-stained liver sections from the control group presented normal histological architecture. Hepatocytes were arranged in radiating cords extending from the central vein and separated by blood sinusoids. The hepatocytes displayed a granular acidophilic cytoplasm and vesicular nuclei with prominent nucleoli. Some hepatocytes were binucleated (Fig. 6a). The MetS group presented numerous hepatocytes with cytoplasmic vacuolations. Some hepatocytes presented deep acidophilic cytoplasm and small dark pyknotic nuclei. A dilated central vein with a thickened wall and prominent inflammatory infiltration was observed (Fig. 6b, c). The MetS + Cranberry 50 group showed almost normal hepatocytes with vesicular nuclei. However, few hepatocytes displayed small, dark pyknotic nuclei and cytoplasmic vacuolation. Mild inflammatory cell infiltration was evident (Fig. 6d). The MetS + Cranberry 100 (Fig. 6e) and metformin (Fig. 6f) groups presented restored normal hepatic architecture with some binucleated cells. Minimal inflammatory cells were observed.

#### PAS reaction

**Fig. 7:**
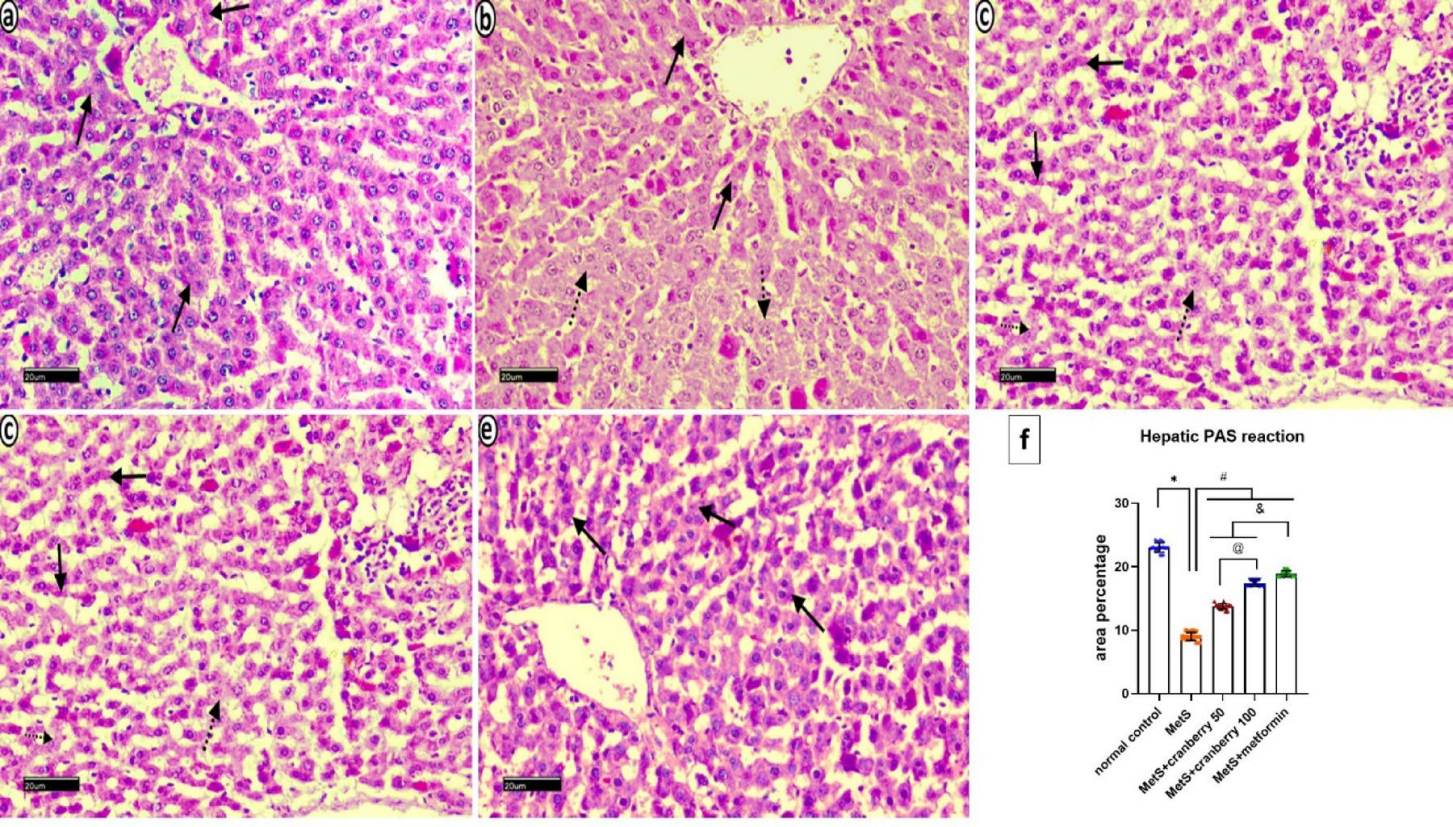
Photomicrographs of PAS-stained liver sections from all the experimental groups. Control group (**a**) reveals normal distribution of glycogen in hepatocytes, with equal reactions across all the hepatic lobules (arrows). The MetS group (**b**) demonstrates many hepatocytes with negative PAS reactions (dotted arrows) and few with positive reactions (arrows). The MetS + Cranberry 50 group (**c**) demonstrated partial restoration of glycogen, with a mixture of PAS-positive (arrows) and negative hepatocytes (dotted arrows). The metS + cranberry 100 (**d**) and metformin (**e**) groups display strong PAS-positive reactions in numerous scattered hepatocytes (arrows). (PAS ×400). (**f**) Liver PAS reaction area percentages in different groups (*n* = 8). P values were considered significant at *p* < 0.05 via ANOVA followed by a post hoc test. *Versus control, ^#^versus MetS, @ versus MetS + Cranberry50, & versus metformin.

Histological analysis via PAS revealed a consistent distribution of glycogen in the hepatocytes of the control group, with even reactions across all the hepatic lobules (Fig. 7a). In contrast, the MetS group presented many hepatocytes with negative PAS reactions and few with positive reactions (Fig. 7b). The MetS + Cranberry 50 group demonstrated a mixture of PAS-positive and -negative hepatocytes (Fig. 7c). The metS + cranberry 100 (Fig. 7d) and metformin (Fig. 7e) groups presented strong PAS-positive reactions in numerous scattered hepatocytes.

#### Masson’s trichrome stain

**Fig. 8:**
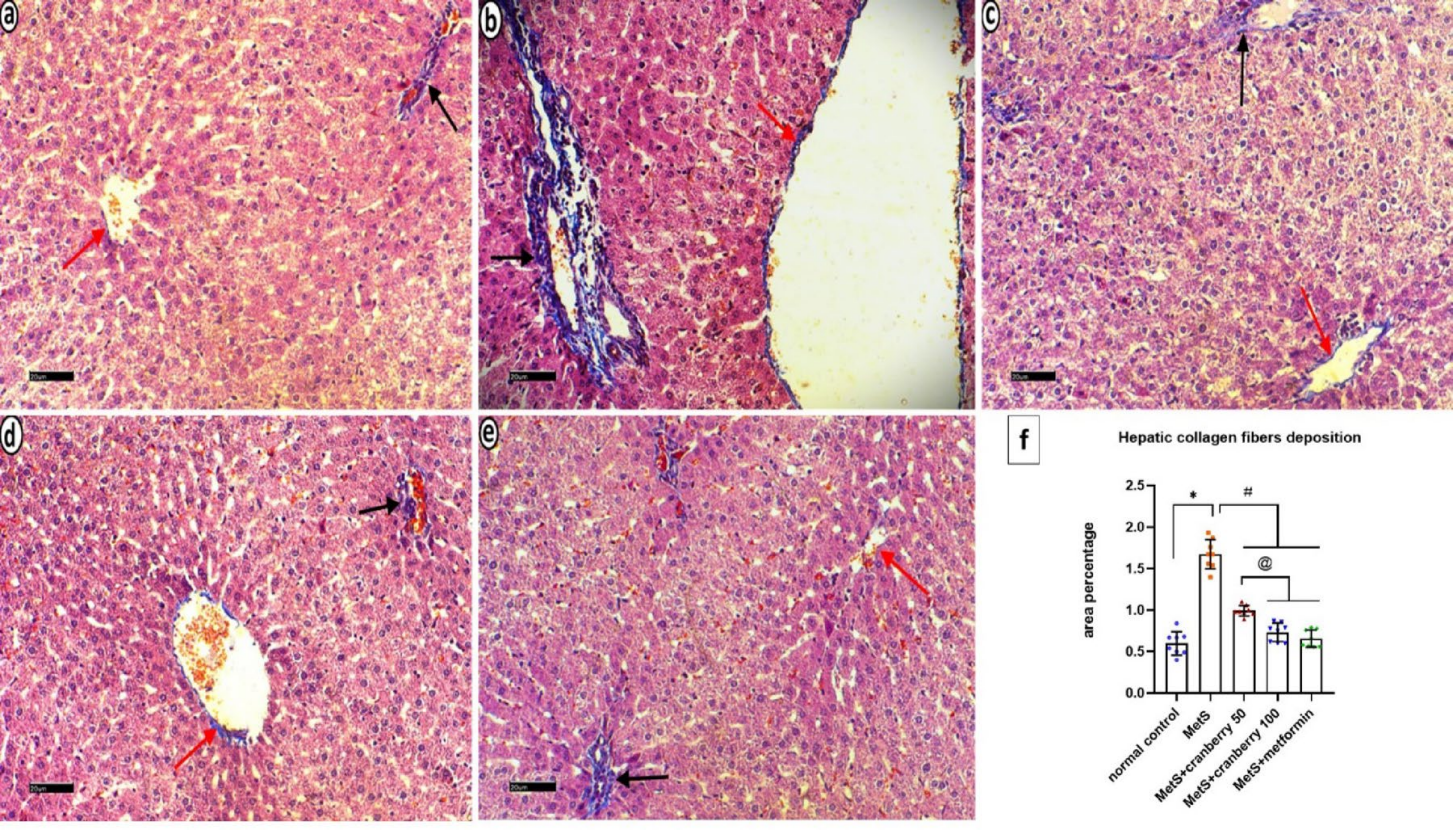
Photomicrographs of liver sections stained with Masson’s trichrome from all experimental groups. Control group (**a**) reveals minimal collagen deposition around the central vein (red arrow) and in the portal area (arrow). The MetS group (**b**) exhibites extensive collagen fiber deposition around the central vein (red arrow) and in the portal area (arrow). MetS + Cranberry 50 group (**c**) showing moderate collagen deposition around the central vein (red arrow) and in the portal area (arrow). The MetS + Cranberry 100 (**d**) and metformin (**e**) groups present mild collagen deposition around the central vein (red arrow) and in the portal area (arrow). (Masson’s trichrome, x400). (**f**) Area percentage of liver collagen fiber deposition in the different groups (*n* = 8). P values were considered significant at *p* < 0.05 via ANOVA followed by a post hoc test. *Versus control, # versus MetS, @ versus MetS + Cranberry 50, & versus metformin.

Masson’s trichrome from the control group revealed minimal collagen deposition around the central vein and in the portal area (Fig. 8a). In contrast, the MetS group exhibited extensive collagen deposition in these regions (Fig. 8b). The MetS + Cranberry 50 group displayed moderate collagen deposition around the central vein and in the portal area (Fig. 8c). The MetS + Cranberry 100 (Fig. 8d) and metformin (Fig. 8e) groups presented mild collagen deposition around the central vein and in the portal area.

### Immunohistochemical staining of iNOS and TGF-β1

**Fig. 9:**
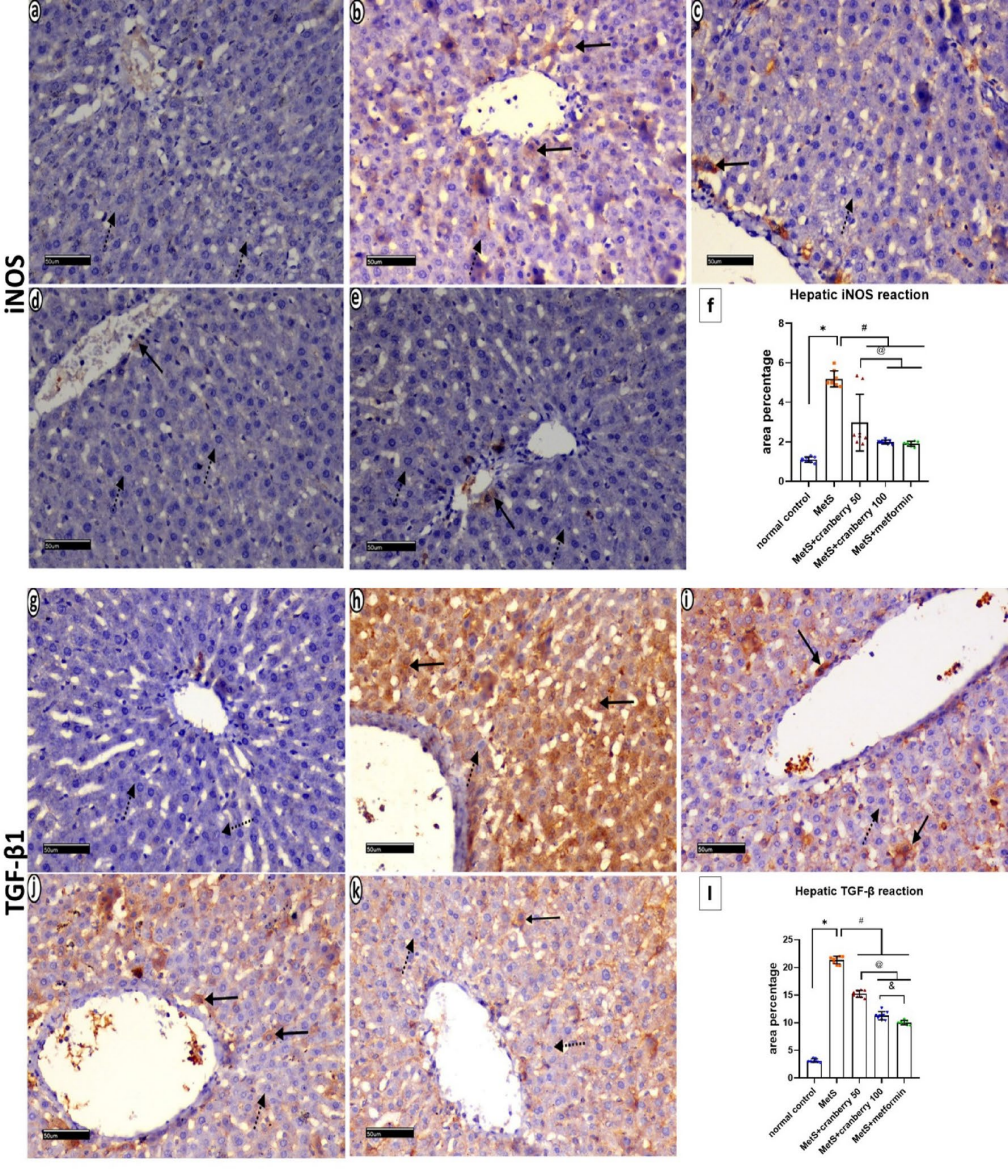
Photomicrographs of immunohistochemical staining of iNOS- and TGF-β1-stained liver sections from all the experimental groups. The control group (**a**,**g**) presents minimal iNOS and TGF-β1 expression (dotted arrows). The MetS group (**b**,**h**) exhibits widespread positive cytoplasmic expression of both iNOS and TGF-β1 in many cells (arrows) and negative expression in a few other cells (dotted arrows). The MetS + Cranberry 50 (**c**,**i**) and MetS + Cranberry 100 (**d**,**j**) groups show a mixed pattern, with some hepatocytes displaying positive expression (arrows) and others negative expression (dotted arrows) of iNOS and TGF-β1. The metformin group (**e**,** k**) demonstrates reduced positive staining for both iNOS and TGF-β1 immunoreactions in few hepatocytes (arrows) and negative expression in many hepatocytes (dotted arrows). (iNOS and TGF-β1 × 200). (**f**,**l**) Representative area percentages of iNOS and TGF-β1 reactions in the different groups (*n* = 8). P values were considered significant at *p* < 0.05 via ANOVA followed by a post hoc test. *Versus control, # versus MetS, @ versus MetS + Cranberry 50, & versus metformin.

Immunohistochemical staining of iNOS and TGF-β1 sections from the control group revealed minimal iNOS (Fig. 9a) and TGF-β1 expression (Fig. 9g). The MetS group exhibited widespread positive cytoplasmic expression of both iNOS (Fig. 9b) and TGF-β1 in many cells (Fig. 9h) and negative expression in few other cells. The MetS + Cranberry 50 and 100 groups showed a mixed pattern, with some hepatocytes displaying positive expression and others negative expression of iNOS (Fig. 9c, d) and TGF-β1 (Fig. 9i, j). The metformin group demonstrated reduced positive staining for both iNOS (Fig. 9e) and TGF-β1 (Fig. 9k), with only few hepatocytes showing positive expression and negative expression in many hepatocytes.

### Kidney

#### Hematoxylin and Eosin

**Fig. 10:**
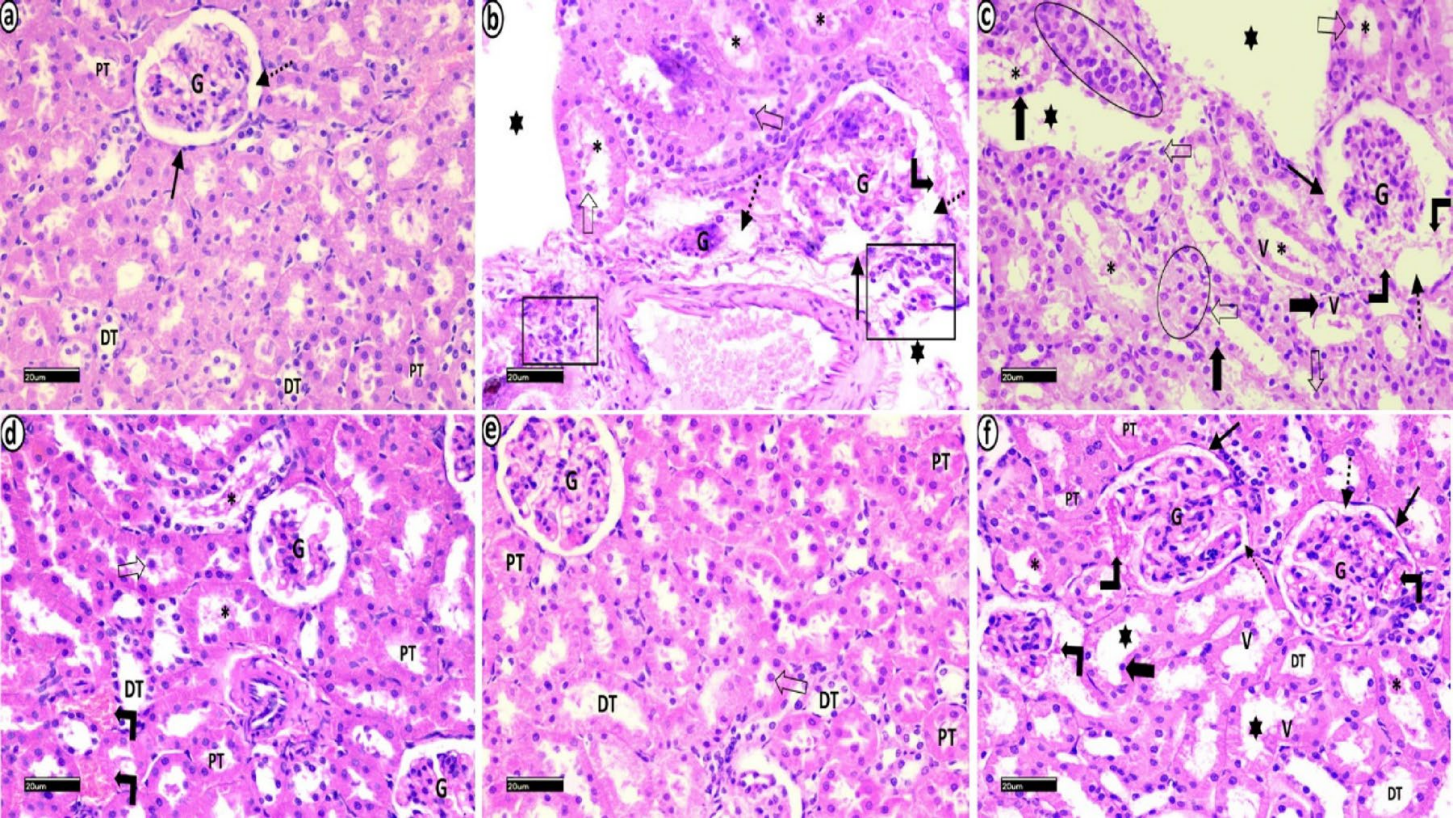
Photomicrographs of H&E-stained sections of the renal cortex from all experimental groups. (**a**) Normal histological architecture of renal corpuscles, formed of Bowman’s capsules lined with simple squamous epithelium (arrow), glomeruli (G) and normal capsular space (dotted arrow), is shown in the control group. The proximal (PT) and distal (DT) tubules have a normal epithelial lining. MetS group (**b**,**c**) showing retraction of glomerular tufts (G) with concomitant widening of the capsular space (dotted arrows). Proximal (PT) and distal (DT) tubules show cystic dilatation (stars), distortion of tubules (black circles), epithelial vacuolations (V), nuclear pyknosis (thick black arrows), epithelial cell detachment (hollow arrows), intraluminal eosinophilic debris (*), inflammatory infiltration (black rectangles) and extravasation of erythrocytes in Bowman’s space (right angled arrows). The MetS + Cranberry 50 group (**d**) shows normal architecture of the glomeruli (G) and apparent normal tubules (PT & DT) but persistent extravasation of erythrocytes (right angled arrows), in between tubules, epithelial cell detachment (hollow arrow), and intraluminal eosinophilic debris (*). MetS + Cranberry 100 group (**e**) showing near normal tubules (PT & DT) with minimal epithelial cell detachment (hollow arrow). The metformin group (**f**) generally presented a normal architecture of renal tissue with occasional extravasation of erythrocytes (right angled arrows), epithelial vacuolations (V), nuclear pyknosis (thick arrow), cystic dilatation (stars) and intraluminal eosinophilic debris (*) (H&E x400).

H&E-stained sections of the renal cortex from the control group revealed normal histological architecture of the renal corpuscles, formed of Bowman’s capsules lined with simple squamous epithelium, glomeruli and a normal capsular space. The proximal and distal tubules had normal epithelial linings (Fig. 10a). The MetS group showed retraction of glomerular tufts with concomitant widening of the capsular space. The proximal and distal tubules exhibited cystic dilatation, distortion of tubules, vacuolations, nuclear pyknosis, epithelial cell detachment, intraluminal eosinophilic debris, inflammatory infiltration and extravasation of erythrocytes in Bowman’s space (Fig. 10b, c). The MetS + Cranberry50 group showed some improvement, with normal glomeruli and tubules but persistent erythrocyte extravasation, epithelial cell detachment, and intraluminal eosinophilic debris (Fig. 10d). The MetS + Cranberry 100 group presented nearly normal tubules with minimal epithelial cell detachment (Fig. 10e). The metformin group generally presented a normal architecture of renal tissue with occasional erythrocyte extravasation, epithelial vacuolations, nuclear pyknosis, cystic dilatation, and intraluminal eosinophilic debris (Fig. 10f).

### Periodic acid schiff (PAS) reaction

**Fig. 11:**
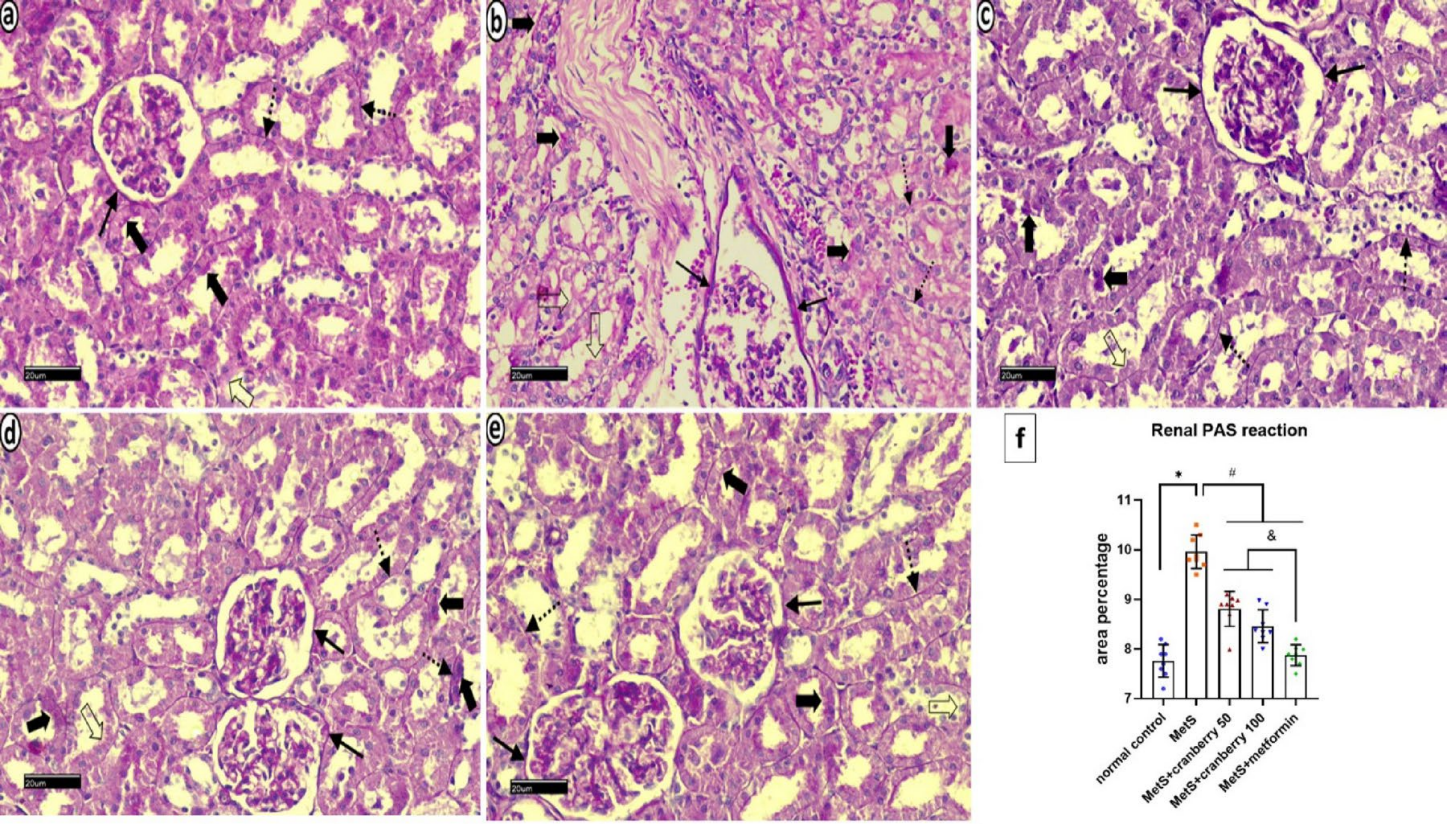
Photomicrographs of PAS-stained sections of the renal cortex from all experimental groups: the control group (**a**) reveales consistent, intense magenta‒red staining of Bowman’s capsule basement membrane (arrow), an intact tubular brush border (thick arrows), and tubular basement membranes (dotted arrows). The metS group (**b**) displays a thickened PAS reaction in Bowman’s capsule basement membranes (arrows), brush border disruption (thick arrows), and lost reaction in the brush border (hollow arrows) of many tubules. There is a faint PAS reaction in the basement membrane of a few tubules (dotted arrows). The MetS + cranberry 50 group (**c**) presentes prominent PAS reaction in basement membrane of Bowman capsule (arrows). Moderate positive PAS reaction in renal tubules brush border (thick arrows) and basement membrane of tubules (dotted arrows). There was no reaction in the luminal borders of few tubules (hollow arrow). The MetS+Cranberry 100 group (**d**) showing moderate positive PAS reaction in basement membrane of Bowman capsule (arrows), along the intact brush border (thick arrows) and regular basement membrane (dotted arrows) in majority of tubules. Few renal tubules show negative luminal brush border PAS reaction (hollow arrow). The metformin group (**e**) showing faint PAS reaction in capsular basement membrane (arrows), intact brush border (thick arrows) and basement membrane (dotted arrows) in majority of renal tubules. There is no reaction in luminal borders of few tubules (hollow arrow) (PAS x400). (**f**) Renal PAS reaction area percentages in different groups (*n* = 8). P values were considered significant at *p* < 0.05 via ANOVA followed by a post hoc test. * versus control, # versus MetS, @ versus MetS + Cranberry 50, & versus metformin.

PAS-stained sections of the renal cortex from the control group revealed consistent, intense magenta‒red reactions of Bowman’s capsule basement membrane, intact tubular brush borders, and tubular basement membranes (Fig. 11a). The metS group displayed a thickened PAS reaction in Bowman’s capsule, brush border disruption, and a diminished reaction in the tubular basement membranes. There was a faint PAS reaction in the basement membrane of a few tubules (Fig. 11b). The metS + Cranberry 50 group presented prominent PAS reaction in basement membrane of Bowman capsule, moderate positive PAS reaction in renal tubules brush border and basement membrane of tubules. There was no reaction in the luminal borders of a few tubules (Fig. 11c). The metS + Cranberry 100 group presented a near-normal PAS reaction, with only few tubules showing a negative luminal border reaction (Fig. 11d). The metformin group exhibited a faint reaction of Bowman’s capsule but a generally intact reaction of the tubular brush border and basement membranes, with occasional interrupted brush borders (Fig. 11e).

### Masson’s trichrome stain

**Fig. 12:**
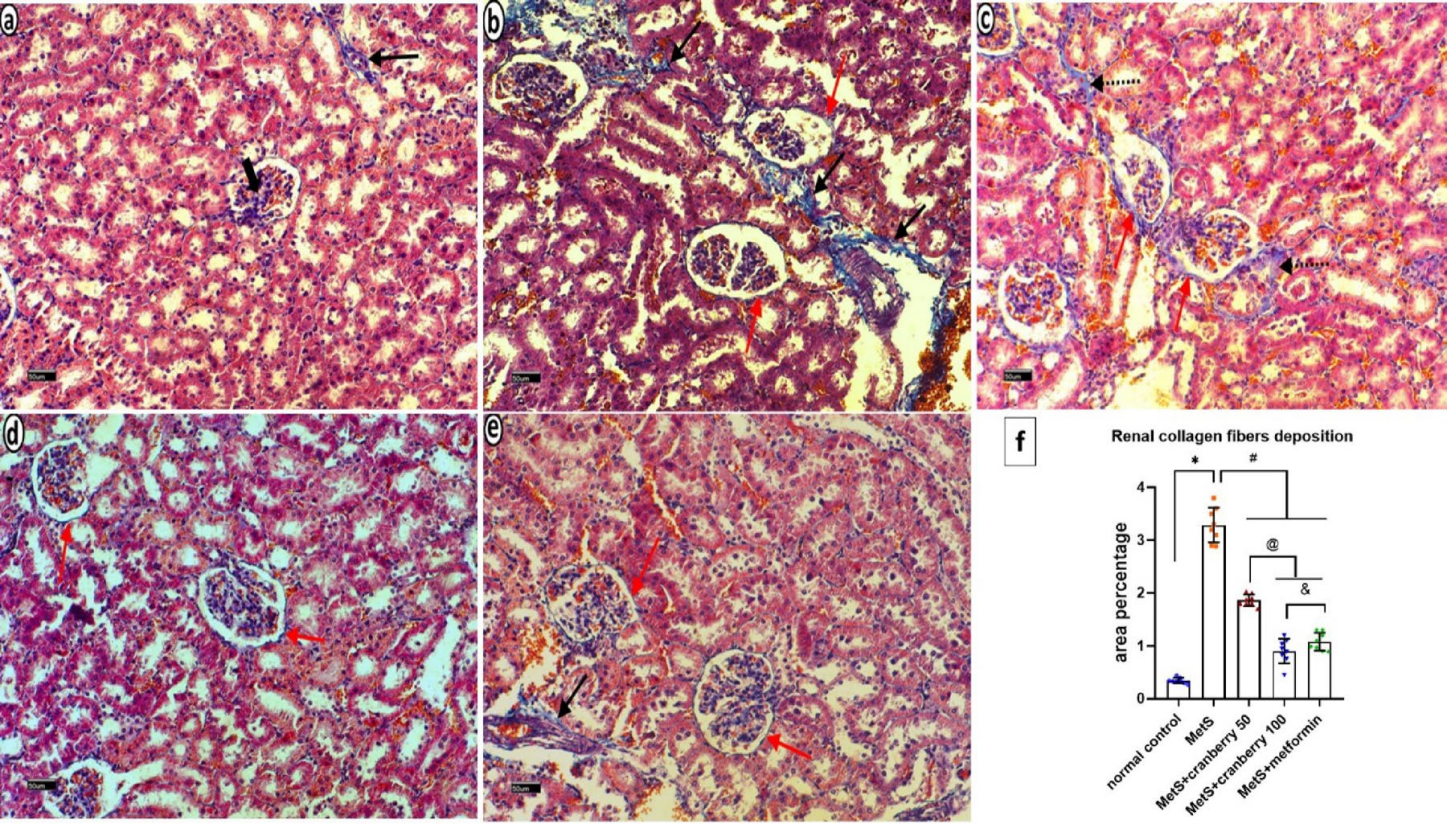
Photomicrographs of kidney sections from all experimental groups stained with Masson’s trichrome. Control group (**a**) Normal glomerular and renal tubules with minimal collagen deposition in the perivascular (arrow) and intraglomerular regions (thick arrow) are shown. The MetS group (**b**) presented extensive collagen deposition, particularly in the perivascular regions (arrows) and Bowman’s capsule (red arrows). MetS + Cranberry 50 (**c**) demonstrates moderate collagen fibers deposition in Bowman’s capsule (red arrows) and between the tubules (dotted arrows). MetS + Cranberry 100 (**d**) exhibits mild deposition in Bowman’s capsule (red arrows). The metformin (**e**) group presented mild collagen deposition in the perivascular region (arrow) and Bowman’s capsule (red arrows). (Masson’s Trichrome ×200). (**f**) Area percentage of renal collagen fiber deposition in the different groups (*n* = 8). P values were considered significant at *p* < 0.05 via ANOVA followed by a post hoc test. *Versus control, # versus MetS, @ versus MetS + Cranberry 50, & versus metformin.

Masson’s trichrome-stained sections of the renal cortex from the control group revealed normal glomerular and renal tubules with minimal collagen deposition in the perivascular and intraglomerular regions (Fig. 12a). The MetS group presented extensive collagen deposition, particularly in the perivascular regions and Bowman’s capsule (Fig. 12b). MetS + Cranberry 50 resulted in a reduction in collagen, with moderate deposition in Bowman’s capsule and between the tubules (Fig. 12c). MetS + Cranberry 100 resulted in a further decrease in collagen, with only mild deposition in Bowman’s capsule (Fig. 12d). The metformin group presented mild collagen deposition in the perivascular region and Bowman’s capsule (Fig. 12e).

### Immunohistochemical staining of iNOS and TGF-β1

**Fig. 13:**
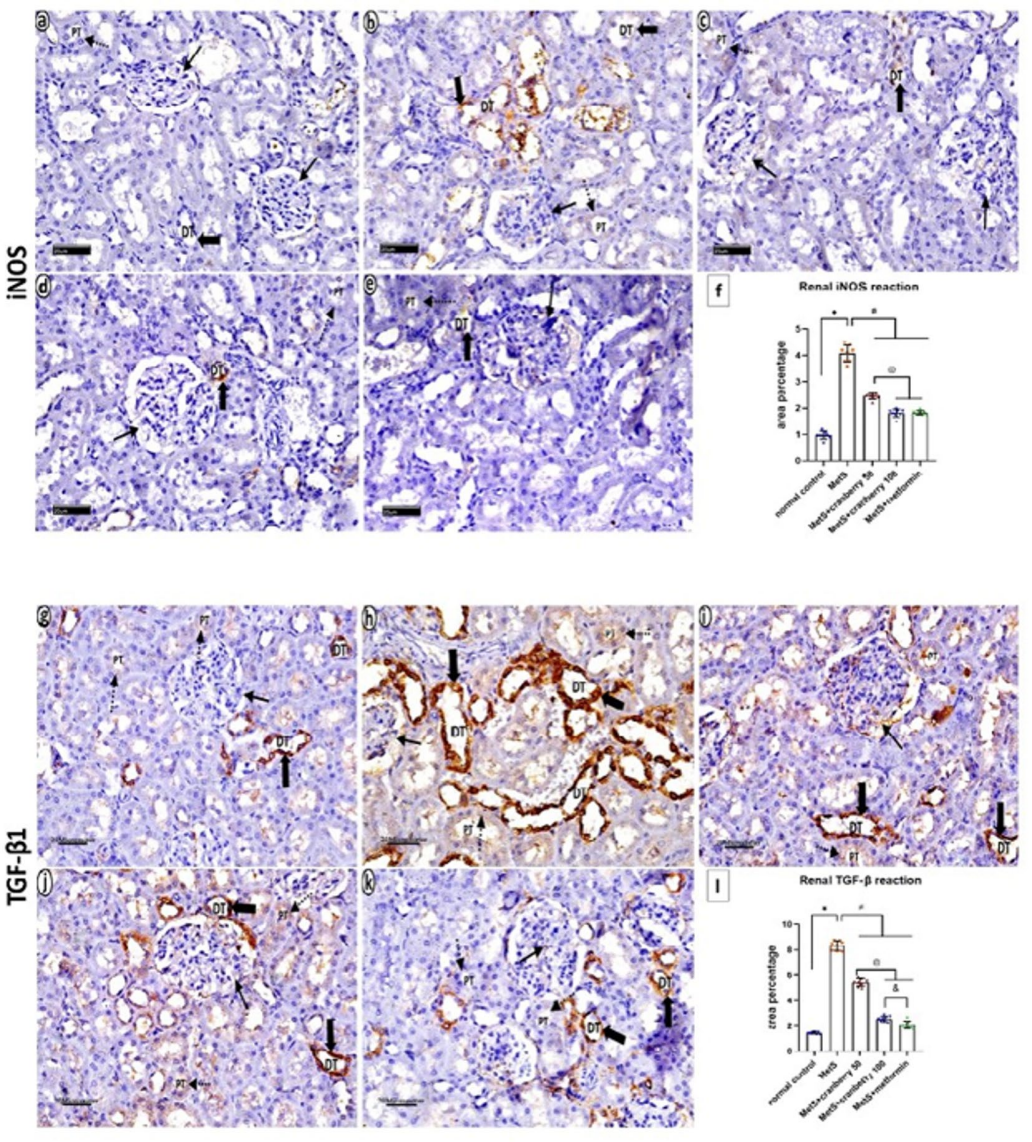
Photomicrographs of immunohistochemical staining of iNOS and TGF-β1 sections from the kidneys of all the experimental groups. Control group (**a**,**g**) reveals negative cytoplasmic expression of iNOS and TGF-β1 in the glomeruli (arrows) and proximal tubules (dotted arrows). Distal tubules appear with negative expression for iNOS and moderate TGF-β1 expression (thick arrows), which appears as a brownish color. The MetS group (**b**,**h**) presented moderate cytoplasmic expression of iNOS and TGF-β1 in the glomeruli (arrows) and proximal tubule tubules (dotted arrows), with extensive expression in the distal tubules which is prominent for TGF-β1 (thick arrows). The MetS + Cranberry50 (**c**,**i**) and MetS + Cranberry100 (**d**,** j**) groups presented mild positive cytoplasmic expression of iNOS and TGF-β1 in the glomeruli (thin arrow) and proximal tubules (dotted arrows) with moderate expression in the distal tubules which is prominent for TGF-β1 (thick arrows). The metformin group (**e**,**k**) presented negative cytoplasmic expression of iNOS in the glomeruli (arrow), proximal tubules (dotted arrow) and distal tubules (thick arrow), whereas TGF-β1 expression was mild in the glomeruli (arrows) and proximal tubules (dotted arrows) and moderate in the distal tubules (thick arrows). (iNOS and TGF-β1 × 400). (**f**,**l**) Representative area percentages of iNOS and TGF-β1 reactions in the different groups (*n* = 8). P values were considered significant at *p* < 0.05 via ANOVA followed by a post hoc test. *Versus control, # versus MetS, @ versus MetS + Cranberry 50, & versus metformin.

Immunohistochemical staining of iNOS and TGF-β1 sections from the control group revealed negative cytoplasmic expression of iNOS (Fig. 13a) and TGF-β1 (Fig. 13g) in the glomeruli and proximal tubules, distal tubules appear with negative reaction for iNOS and moderate TGF-β1 expression which appeared as a brownish color. The MetS group presented moderate cytoplasmic expression of iNOS (Fig. 13b) and TGF-β1 (Fig. 13h) in the glomeruli and proximal tubules, with extensive expression in the distal tubules which is prominent for TGF-β1. The MetS + Cranberry 50 and MetS + Cranberry 100 groups presented mild positive cytoplasmic expression of iNOS (Fig. 13c, d) and TGF-β1 (Fig. 13i, j) in the glomeruli and proximal tubules with moderate TGF-β1 expression in the distal tubules. The metformin group presented negative iNOS expression throughout the kidney (Fig. 13e), while TGF-β1 expression was mild in the glomeruli and proximal tubules and moderate in the distal tubules (Fig. 13k).

### Morphometric results

#### Mean area percentage of the PAS reaction

In the liver, a significant decrease in the mean area % of the PAS reaction was detected in the MetS group compared with the control group. The MetS + Cranberry 50, 100, and metformin groups presented significantly greater PAS reactions than the MetS group. However, both the MetS + Cranberry groups presented lower PAS reactions than the metformin group, with the MetS + Cranberry 50 group showing the lowest reaction among the treatment groups (≤ 0.05) (Fig. 7f).

In the kidney, a significant increase in the mean area percentage of the PAS reaction was detected in the MetS group compared with the control group. The MetS + Cranberry 50, 100, and metformin groups presented significantly lower PAS reactions than the MetS group. However, both the MetS + Cranberry groups presented significantly greater PAS reactions than the metformin group. (*P* ≤ 0.05) (Fig. 11f).

#### Mean area percentage of collagen fibers deposition  

In the liver, a significant increase in the mean area percentage of Masson’s Trichrome stain was detected in the MetS group compared with the control group. The MetS + Cranberry 50, 100, and metformin groups presented significantly lower mean area percentages of collagen fibers deposition when compared with the MetS group. There was a nonsignificant increase in the MetS + Cranberry 100 group compared with the metformin group. There was a nonsignificant difference between the MetS + Cranberry 100 and metformin groups and the control group (*P* ≤ 0.05) (Fig. 8f).

In the kidney, a significant increase in the mean area percentage of collagen fibers deposition was detected in the MetS group compared with the control group. A significant decrease in mean area percentage of collagen fibers deposition in MetS+Cranberry 50, MetS+Cranberry 100 and Metformin groups as compared to MetS group. A significant decrease was observed between both of MetS+Cranberry 100 and Metformin as compared with the MetS+Cranberry 50 group. There was a significant decrease in MetS+Cranberry 100 group as compared to Metformin group (*P* ≤ 0.05) (Fig. 12f).

#### Mean area percentage of iNOS immunoreactivity

In the liver, there was a significant increase in the mean area collagen fibers deposition of iNOS immunoreaction in the MetS group compared with the control group. Moreover, the MetS + Cranberry 50, 100, and metformin groups presented significantly lower iNOS expression than the MetS group. There was a nonsignificant difference between the MetS + Cranberry 100 and metformin groups, and neither were significantly different from the control (*P* < 0.05). (Fig. 9f).

In the kidney, the mean percentage area of iNOS immunoreactivity in the MetS group was significantly greater than that in the control group. Moreover, the MetS + Cranberry 50, 100, and metformin groups presented significantly lower mean area percentages of iNOS expression than the MetS group. The MetS + Cranberry 50 and metformin groups presented significantly lower mean area percentages of iNOS expression when compared with the MetS+Cranberry 100 and metformin groups. There was a nonsignificant difference between the MetS + Cranberry 100 and metformin groups (*P* < 0.05). (Fig. 13f).

#### Mean area percentage of TGF-β1 immunoreactivity

In the liver, there was a significant increase in the mean area percentage of the TGF-β1 immunoreaction in the MetS group compared with that in the control group. Meanwhile, a significant decrease in the mean area percentage of TGF-β1 in MetS+Cranberry 50, MetS+Cranberry 100 and Metformin groups as compared to MetS group. Moreover, the MetS + Cranberry 100 and metformin groups presented significantly lower mean areas of TGF-β1 expression than did the MetS + Cranberry 50 group. Compared with the metformin group, the MetS + Cranberry 100 group presented a significantly greater mean area % of TGF-β1 expression (*P* < 0.05) (Fig. 9L).

In the kidney, there was a significant increase in the mean area percentage of the TGF-β1 immunoreaction in the MetS group compared with that in the control group. Moreover, the MetS + Cranberry 50, 100, and metformin groups presented significantly lower mean area percentages of TGF-β1 expression than did the MetS group. A significant decrease was observed between both of MetS+Cranberry 100 and Metformin as compared with the MetS+Cranberry 50 group. Compared with the metformin group, the MetS + Cranberry 100 group presented significantly higher TGF-β1 expression (*P* < 0.05) (Fig. 13L).

To summarize the histological and morphometric results, MetS induction led to significant organ damage in the liver and kidneys, characterized by cellular injury, extensive collagen deposition, and increased immunoreactivity for iNOS and TGF-β1. Both cranberry extract, in a dose-dependent manner, and metformin substantially mitigated these pathological changes, leading to a restoration of more normal tissue architecture, a significant reduction in fibrosis, and a decrease in both iNOS and TGF-β1 immunoexpression.

## Discussion

Metabolic syndrome is not a disease in itself but rather a cluster of interconnected risk factors that significantly increase the likelihood of developing serious health problems. This syndrome is becoming increasingly prevalent globally and represents a major public health concern. The following discussion delves more deeply into the multifaceted protective actions of cranberry vs. metformin on hepatic and renal health in a metabolic syndrome rat model, exploring the interconnected mechanisms through which both interventions modulate key pathways, such as the AMPK, RhoA/ROCK, and Srebf1 pathways, to improve metabolic function, reduce fibrosis, and ultimately preserve organ integrity.

The present study induces MetS using a high-fat/high-fructose diet combined with low-dose streptozotocin (STZ), this model mimics human metabolic syndrome by inducing key features such as insulin resistance, dyslipidemia, and mild hyperglycemia. The diet promotes obesity and metabolic imbalance, while STZ partially impairs β-cell function, replicating early type 2 diabetes. This model also reflects associated organ dysfunction, making it suitable for studying MetS pathophysiology and interventions.

The occurrence of MetS evidenced by significantly increased body weight, BMI, fasting glucose levels, glucose tolerance, and systolic and diastolic blood pressure compared with those of the normal control group. In addition, lipid profile alterations are characteristic of MetS-induced dyslipidemia. These findings are consistent with previous studies that have shown similar metabolic alterations in metabolic syndrome models^31^.

Treatment with cranberry extract (at doses of 50 and 100 mg/kg) significantly reduced these parameters, and these findings were comparable with those of metformin treatment.

Cranberry extract reduced diet-induced weight gain and visceral fat accumulation and improved insulin sensitivity secondary to decreased inflammation and OS, in accordance with previous studies^32^. The lipid-lowering effects of cranberry extract may be attributed to its polyphenolic content, which can modulate lipid metabolism and reduce OS^8^. In accordance with Liu (2024)^33^ metformin improved MetS-induced fasting glucose, TG, TC, LDL-C, systolic and diastolic blood pressure and waist circumference through improving vascular endothelial function and OS.

The lipid-lowering effects of cranberry or metformin can be linked to the increased expression of AMP-activated protein kinase (AMPK). The activation of AMPK inhibits lipogenesis and promotes fatty acid oxidation^34^. AMPK is a crucial regulator of energy homeostasis, and its activation has been shown to improve insulin sensitivity and reduce lipid accumulation^35^. Previous studies reported that polyphenols improve AMPK expression in a therapeutic approach for hyperlipidemia ^36 37^.

The dose-dependent AMPK upregulation by cranberry 100 mg/kg surpassing metformin is a particularly noteworthy finding. Cranberry extract is rich in a diverse array of polyphenols, including various flavonoids (proanthocyanidins, anthocyanins, flavonols, etc.). Certain polyphenols are known to activate Sirtuin 1 (SIRT1) that is an upstream activator of AMPK. The cranberry’s diverse polyphenols synergistically activate AMPK, potentially through robust engagement of the SIRT1-AMPK axis, or by achieving a critical bioavailability threshold for key compounds at the higher dose.

In the present study, the MetS group presented significantly increased Srebf1 expression, which was reduced by cranberry or metformin treatment. SREBP-1c activates the transcription of genes involved in fatty acid and triglyceride synthesis^38^. Inhibition of SREBP-1c, a downstream consequence of AMPK activation, reduce its nuclear translocation and transcriptional activity^39^.

The MetS group presented significantly elevated ROCK1 expression, which was reduced by all the treatments. These findings indicate that the inhibition of ROCK1 may contribute to the metabolic improvements observed with cranberry extract and metformin. Lee et al.. reported that the ROCK/AMPK/SREBP-1c pathway plays a key role in regulating cellular lipid accumulation and preventing lipotoxicity^40^.

The fasting blood glucose level was significantly greater in the MetS group than in the normal control group, and the glucose tolerance test further confirmed disrupted glucose homeostasis in diabetic MetS rats. This presents significantly higher blood glucose levels at multiple time points in the MetS group than in the normal group. Treatment with cranberry extract as well as the standard anti-diabetic drug metformin significantly improved these levels, resulting in glucose levels closer to those of the normal group and a significantly lower glucose AUC values compared to the untreated MetS group. These findings align with previous research demonstrating the glucose-lowering effects of cranberry extract^8^.

A cornerstone of metformin’s action is the activation of AMPK by modulating cellular energy status via mitochondrial interference^41^ which subsequently inhibits hepatic gluconeogenesis^42^. and enhances glucose uptake in peripheral tissues via GLUT4 translocation^43^. This offers a clear mechanistic explanation for the enhanced glucose disposal seen in the OGTT, as well as the replenishment of hepatic glycogen stores at the tissue level as visualized with PAS staining.

It is noteworthy that the mechanisms through which cranberry extract appears to exert its benefits closely parallel those of the positive control, metformin. Crucially, our results indicate that cranberry extract may be even more effective in this regard, as the 100 mg/kg dose induced a significantly greater upregulation of AMPK expression compared to metformin, suggesting a robust effect on cellular bioenergetics, potentially supplemented by other upstream kinases like LKB1 and SIRT1^44^. This positions cranberry extract not merely as a functional parallel to metformin, but as a potentially more potent activator of this central metabolic sensor.

While serum insulin and HOMA-IR was not directly measured in this study, the marked improvement in the OGTT results, coupled with the robust activation of the AMPK pathway, strongly suggests that the therapeutic effects of cranberry extract are mediated, at least in part, by an enhancement of whole-body insulin sensitivity.

Furthermore, the inhibition of ROCK1 is increasingly recognized for its beneficial effects on glucose metabolism. Elevated ROCK1 activity contributes to insulin resistance, and its inhibition, as seen in our treatment groups, has been shown to improve insulin sensitivity and directly promote GLUT4 translocation in insulin-responsive tissues^45–47^.

Therefore, the concurrent downregulation of ROCK1 and upregulation of AMPK by cranberry extract points to a sophisticated and interconnected regulatory effect on glucose metabolism, and provides a powerful explanation for the significant improvements observed in our functional glucose assays, including the enhanced glucose tolerance and the restoration of hepatic glycogen stores.

The comparable efficacy of cranberry extract and metformin in reducing body weight, BMI, and fasting glucose levels suggests that cranberry extract could be a viable alternative or adjunct to conventional antidiabetic medications.

Metabolic syndrome is frequently associated with hypertension, increasing the risk of cardiovascular complications. Treatment with either cranberry extract or metformin had significant antihypertensive effects, as evidenced by the reductions in the SBP, DBP, and MAP. These findings align with those of previous studies^33,48–50^ which documented the blood pressure-lowering properties of both interventions.

A plausible mechanism for this effect involves the modulation of nitric oxide (NO) bioavailability, a critical regulator of vascular tone. In the pathological state of MetS, the upregulation of inducible nitric oxide synthase (iNOS), as observed in our study, can be detrimental. As reported by Magdy et al.^51^when iNOS-driven NO production is excessive, it readily reacts with superoxide anions (O₂⁻) to form peroxynitrite (ONOO⁻). Peroxynitrite is a highly reactive species that promotes endothelial dysfunction and impairs vasodilation rather than improving it. By potentially dampening iNOS expression in blood vessels, as that observed in the present hepatic and renal tissues, cranberry extract appears to interrupt this damaging pathway, which is consistent with studies reporting that its constituent polyphenols exert strong antioxidant effects^52^and can improve endothelial function by preserving beneficial eNOS-derived NO signaling^53^. Furthermore, the observed AMPK activation in our study has also been shown to activate eNOS and enhance NO bioavailability^54^. Therefore, the antihypertensive action of cranberry extract is likely mediated through a dual mechanism: suppressing detrimental iNOS-derived pathways while promoting beneficial eNOS-derived vasodilation.

Furthermore, earlier studies have established a mechanistic link between AMPK activation and RhoA/ROCK suppression with blood pressure regulation, as demonstrated by Cao et al.^55^. The observed AMPK activation and RhoA/ROCK1 suppression in our study may therefore have contributed to the blood pressure-lowering effects of our interventions.

The concurrent downregulation of ROCK1 and upregulation of AMPK by cranberry extract, as observed in our study, points to a sophisticated regulatory effect. This interplay is likely pivotal in the amelioration of metabolic syndrome-related organ dysfunction. Cranberry polyphenols, such as proanthocyanidins, anthocyanins, and flavonols, may modulate these pathways through several interconnected mechanisms. Firstly, these phytoconstituents are hypothesized to directly activate AMPK via established upstream kinases like LKB1& SIRT1 by engaging the SIRT1-AMPK axis^44^. Secondly, cranberry components may independently inhibit the RhoA/ROCK1 pathway, by directly inhibiting ROCK kinase expression. There is also emerging evidence of direct crosstalk, where AMPK activation itself can negatively regulate RhoA/ROCK signaling^55^suggesting that cranberry-induced AMPK upregulation might also contribute to the observed ROCK1 inhibition. Furthermore, the potent antioxidant and anti-inflammatory properties of cranberry polyphenols can create a cellular environment that both favors AMPK activation and suppresses ROCK1 signaling, potentially by mitigating common upstream stressors like oxidative stress.

Thus, cranberry extract appears to orchestrate a beneficial shift by both independently targeting these pathways and enhancing their intrinsic regulatory connections, leading to reduced lipogenesis, inflammation, fibrosis, and improved metabolic homeostasis.

In summary, AMPK activation is strongly implicated as a primary driver of many beneficial effects. As a central metabolic sensor, its activation by cranberry polyphenols (potentially via upstream kinases like LKB1), or indirectly through effects on cellular energy status or SIRT1 activation, directly leads to first enhanced glucose uptake and fatty acid oxidation. Second, inhibition of SREBP-1c, a downstream consequence of AMPK activation, thereby reducing lipogenesis. And potential indirect suppression of ROCK1 activity, as some studies suggest crosstalk where AMPK can negatively regulate RhoA/ROCK signaling.

The physiological and biochemical derangements in the MetS group were mirrored by profound histological damage in both hepatic and renal tissues, consistent with previous studies^56,57^. H&E staining of the liver revealed extensive pathological alterations, including hepatocyte vacuolation, pyknotic nuclei, and inflammatory infiltration. The kidneys exhibited equally significant injury, characterized by glomerular retraction, cystic tubular dilatation, and epithelial cell detachment. These structural changes were accompanied by disruptions in cellular metabolism. Hepatic PAS staining demonstrated a marked depletion of glycogen stores in the MetS group consistent with previous studies^11^, indicating a fundamental disruption of glucose homeostasis linked to the impaired AMPK signaling discussed earlier. In the kidneys, PAS staining revealed pathological alterations, including a thickened Bowman’s capsule basement membrane and disruption of the tubular brush border, features consistent with diabetic nephropathy^58^.

A key molecular driver of the observed hepatic and renal injury in the MetS group is the significant upregulation of inducible nitric oxide synthase (iNOS). Our study confirmed this, revealing markedly elevated iNOS immunoexpression in the liver and kidney tissues of MetS rats, which correlated with the observed cellular damage. While NO is a critical signaling molecule, excessive production by iNOS becomes cytotoxic. As reported by Magdy et al.^51^this excess NO produces a highly reactive nitrogen species and a potent mediator of cellular damage that promotes oxidative stress, lipid peroxidation, and apoptosis. This mechanism provides a strong explanation for the histological damage, including pyknosis and vacuolation, seen in the untreated MetS group. Therefore, the ability of cranberry extract and metformin to markedly dampen iNOS expression represents a crucial hepatoprotective and nephroprotective mechanism.

The present study revealed several key indicators of fibrosis in both the hepatic and renal tissues of MetS rats. Histopathological examination revealed significant collagen fiber accumulation, particularly around the central vein and in portal areas in the liver and in the perivascular regions and Bowman’s capsule in the kidney, as shown by Masson’s trichrome staining.

Furthermore, the present results revealed increased immunoexpression of hepatic and renal TGF-β1. The increased production of TGF-β1 by hepatocytes and Kupffer cells plays a crucial role in extracellular matrix synthesis and deposition, potentially inducing both fibrogenesis and hepatocyte apoptosis. This finding is consistent with those of previous studies ^59,60^. Apoptotic hepatocytes release damage-associated molecular patterns (DAMPs)^61^which further activate Kupffer cells and hepatic stellate cells (HSCs), creating a vicious cycle of inflammation and fibrosis^62^.

Similarly, the initial alteration in the pathogenesis of diabetic nephropathy is the expansion of the glomerular mesangium due to excessive extracellular matrix (ECM) protein accumulation. TGF‑β plays a key role in this process, influencing cell proliferation, differentiation, apoptosis, autophagy and ECM production^63^. The overexpression of TGF‑β may induce epithelial‑mesenchymal transition (EMT) and renal sclerosis^64^.

Our results demonstrated that treatment with both cranberry extract and metformin effectively decreased the immunoexpression of TGF-β1 in the liver and kidney of MetS rats. This observation aligns with that of Faheem et al.^65^. who also highlighted the modulation of TGF-β1 in the context of the hepatoprotective effects of cranberries.

Furthermore, the consistent upregulation of ROCK1 and TGF-β1 in our metabolic syndrome model and their modulation by cranberries reinforce their synergistic roles in fibrotic processes. Previous studies have revealed a direct link between TGF-β1 and ROCK1 in HSC-mediated fibrosis^66^. Furthermore, the RhoA/ROCK pathway, which includes both ROCK1 and ROCK2 isoforms, is implicated in HSC contraction and activation, further supporting its central role in liver fibrogenesis ^67–69^.

Similarly, Masumoto et al. reported the profibrotic roles of ROCK isoforms and TGF-β1 in the kidney^70^. ROCK1 knockout has been demonstrated to ameliorate albuminuria in diabetic kidney disease^71^.

In summary, the downregulation of ROCK1 expression by cranberry is likely a primary effect contributing to reduced inflammation, fibrosis and improved insulin sensitivity. Polyphenols are known to modulate pathways that can influence ROCK activity^72^. Its inhibition would then contribute to ameliorating MetS features.

The modulation of TGF-β1 is likely a combination. Elevated TGF-β1 in MetS contributes to fibrosis and inflammation. Cranberry’s ability to reduce TGF-β1 could be a primary effect (e.g., antioxidant actions reducing TGF-β1 induction) or secondary to reduced tissue damage, inflammation (e.g., via ROCK1 inhibition or improved metabolic health), which then lessens the stimulus for TGF-β1 production. The interplay between ROCK1 and TGF-β1 in fibrotic processes suggests they can influence each other.

The present results suggest potential clinical applications for treating metabolic syndrome, with cranberry extract as an alternative for treating metformin-intolerant patients. Owing to their natural origin and multiple protective mechanisms, cranberries are attractive for preventive strategies and long-term use, suggesting promising therapeutic potential for metabolic syndrome and related liver and kidney damage.

In conclusion, the present study highlighted that cranberry extract emerges as a promising candidate. The rationale for its use extends beyond simply being an alternative; it lies in its distinct, multi-target mechanism and favorable safety profile. Our findings indicate that cranberry extract not only matches metformin’s efficacy in improving glycemic control and lipid profiles but may offer broader benefits. By concurrently suppressing the pro-inflammatory and pro-fibrotic ROCK1 and TGF-β1 pathways, downregulating the lipogenic SREBP-1c pathway, and more potently activating the central metabolic regulator AMPK, cranberry extract provides a more comprehensive approach to ameliorating the multifaceted organ dysfunction characteristic of MetS. Therefore, cranberry extract could serve as a valuable alternative for metformin-intolerant patients or as an adjunctive therapy on both metabolic control and end-organ protection.

### Limitations and future perspectives

We acknowledge that the 4-week treatment duration, while sufficient to observe significant effects in this rat model of MetS, may not fully reflect the chronic nature of metabolic syndrome in humans or the long-term impact of cranberry supplementation. Consequently, long-term efficacy and safety data for cranberry extract in the context of MetS management in humans represent an important area for future clinical investigation.

## Data Availability

The datasets used and/or analyzed during the current study are all available in the current manuscript.

## Declarations

### Prior publication

The data presented in this manuscript have not been published previously.
